# Novel insights into the mechanism(s) of silicon-induced drought stress tolerance in lentil plants revealed by RNA sequencing analysis

**DOI:** 10.1186/s12870-023-04492-5

**Published:** 2023-10-17

**Authors:** Sajitha Biju, Sigfredo Fuentes, Dorin Gupta

**Affiliations:** School of Agriculture, Food and Ecosystem Sciences (SAFES), Faculty of Science, The University of Melbourne, Parkville, VIC 3010 Australia

**Keywords:** Carbohydrates, Cell wall biosynthesis, Differential genes expression, Hormones, Lipids, Osmo protection, Photosynthesis, Proteins, Reactive oxygen species, Secondary metabolites, Vasculature

## Abstract

**Background:**

Lentil is an essential cool-season food legume that offers several benefits in human nutrition and cropping systems. Drought stress is the major environmental constraint affecting lentil plants’ growth and productivity by altering various morphological, physiological, and biochemical traits. Our previous research provided physiological and biochemical evidence showing the role of silicon (Si) in alleviating drought stress in lentil plants, while the molecular mechanisms are still unidentified. Understanding the molecular mechanisms of Si-mediated drought stress tolerance can provide fundamental information to enhance our knowledge of essential gene functions and pathways modulated by Si during drought stress in plants. Thus, the present study compared the transcriptomic characteristics of two lentil genotypes (drought tolerant-ILL6002; drought sensitive-ILL7537) under drought stress and investigated the gene expression in response to Si supplementation using high-throughput RNA sequencing.

**Results:**

This study identified 7164 and 5576 differentially expressed genes (DEGs) from drought-stressed lentil genotypes (ILL 6002 and ILL 7537, respectively), with Si treatment. RNA sequencing results showed that Si supplementation could alter the expression of genes related to photosynthesis, osmoprotection, antioxidant systems and signal transduction in both genotypes under drought stress. Furthermore, these DEGs from both genotypes were found to be associated with the metabolism of carbohydrates, lipids and proteins. The identified DEGs were also linked to cell wall biosynthesis and vasculature development. Results suggested that Si modulated the dynamics of biosynthesis of alkaloids and flavonoids and their metabolism in drought-stressed lentil genotypes. Drought-recovery-related DEGs identified from both genotypes validated the role of Si as a drought stress alleviator. This study identified different possible defense-related responses mediated by Si in response to drought stress in lentil plants including cellular redox homeostasis by reactive oxygen species (ROS), cell wall reinforcement by the deposition of cellulose, lignin, xyloglucan, chitin and xylan, secondary metabolites production, osmotic adjustment and stomatal closure.

**Conclusion:**

Overall, the results suggested that a coordinated interplay between various metabolic pathways is required for Si to induce drought tolerance. This study identified potential genes and different defence mechanisms involved in Si-induced drought stress tolerance in lentil plants. Si supplementation altered various metabolic functions like photosynthesis, antioxidant defence system, osmotic balance, hormonal biosynthesis, signalling, amino acid biosynthesis and metabolism of carbohydrates and lipids under drought stress. These novel findings validated the role of Si in drought stress mitigation and have also provided an opportunity to enhance our understanding at the genomic level of Si’s role in alleviating drought stress in plants.

## Background

Lentil is an important cool season food legume crop and is sensitive to drought stress, especially during anthesis with devastating effects on production and yield [1–4]. Responses of lentil towards drought stress have been studied, based on various agronomical, morphological, physiological, biochemical, and molecular approaches [2, 5–11]. However, an appropriate and sustainable management strategy to maintain crop performance under drought is still lacking. Therefore, identifying a suitable stress alleviator to promote sustainable drought management is essential for lentil adaptation in adverse environments.

Silicon enhances plant growth, yield and tolerance to various environmental stresses and thus gained attention as an essential element in agriculture [12–14]. Silicon alleviates drought stress in both monocot and dicot plants by modulating physiological and biochemical mechanisms [12, 15–19], but the underlying molecular mechanisms of its effect remain poorly understood. In plants, Si modulates complex metabolic activities including, photosynthesis, osmotic adjustments, antioxidant metabolism, phytohormonal interactions and metabolism related to protein, carbohydrates, lipids, and secondary metabolites under abiotic stress environments [20–24]. The ameliorative effects of Si on abiotic stresses have been investigated at the molecular levels in plants [25–32]. Silicon nanoparticles (SiNp) modulated the expression of salt stress genes in salinity-stressed tomato seedlings by enhancing the expression of abscisic acid responsive element-binding protein (AREB), abscisic acid and environmental stress-inducible protein (TAS14), 9-cis-epoxycarotenoid dioxygenase (NCED30 and cysteine-rich receptor-like protein kinase genes (CRK1) and decreasing the expression of respiratory burst oxidase (RBOH1), cytosolic ascorbate peroxidase (APX2), mitogen-activated protein kinase (MAPK2), ethylene response factor (ERF5) dwarf and delayed flowering genes (DDF2) [33]. These up- or downregulated genes are involved in regulating the responses of abscisic acid (ABA), and the activation of the antioxidant defense system in plants under stress. Silicon-mediated regulation of the expression of genes *Csa3G199590* (transcription factor MYB44-like), *Csa6G091830* (AP2 domain transcription factor RAP2), *Csa1G033310* (Chlorophyll a-b binding protein P4, chloroplastic-like), *Csa6G104650* (auxin-induced protein 5NG4-like gene) and *Csa2G000790* (oxidative stress) have contributed to enhanced salt tolerance in cucumber plants. These genes regulate photosynthesis, oxidative stress and auxin signaling pathway in cucumber plants under stress [34]. Silicon-mediated alleviation of Cd toxicity is reported in rice and wheat plants with alterations in the expression patterns of Cd transporter genes (*OsNramp5, OsHMA2, TaNramp5, TaTM20* and *TaHMA3*) responsible for Cd uptake and translocation [35–37]. However, from a molecular perspective, limited studies have been published to explore the mechanisms of Si-mediated drought stress tolerance in plants. In rice, Si application mitigated drought stress through enhanced expression of the transcription factors, dehydration-responsive element-binding protein *DREB2A*, and *NAC5* [no apical meristem (NAM), *Arabidopsis thaliana* activating factor (ATAF), and cup-shaped cotyledon (CUC)], which control various defence pathways involved in drought stress tolerance [38]. Silicon application increased the relative expression levels of genes encoding antioxidant enzymes in the ascorbate glutathione cycle (*TaSOD, TaCAT, TaAPX, TaGR, TaDHAR, TaMDHAR*, and *TaGS*) and restored the gene expressions of *TaPAL, TaCHS, TaF3H, TaDFR, and TaANS,* which encode enzymes involved in the flavonoid biosynthesis pathway, in drought-stressed wheat plants [39]. Most recently, Boora et al. [40] reported that the application of biosynthesised SiNPs could mitigate drought stress in wheat plants through the upregulation of stress-related genes such as *DREB2 (*dehydration response), *TaMYB33* (osmotic equilibrium recovery and ROS detoxification), *WRKY 19* (hormone signaling, secondary metabolite biosynthesis), and *SnRK* (ROS scavenging and ABA-dependent signal transduction).

Silicon supplementation is also known to modulate the signalling mechanisms of one or more phytohormones such as abscisic acid (ABA), gibberellic acid (GA), jasmonic acid (JA), brassinosteroid (BR), ethylene (ET), salicylic acid (SA), cytokinin (CK) and auxin (AUX) by overexpressing the genes that control their production under abiotic stress in plants [41–45]. Silicon significantly boosted the expression of AUX biosynthesis genes, OsYUCCA1 and OsTAA1 in rice plants under arsenate stress [46], and regulated the expression of genes in CK signalling pathway (*Csa4G647490, Csa1G589070, Csa7G392940* and *Csa3G150100*) in salt-stressed cucumber plants [34] and downregulated the expression levels of ABA biosynthetic genes (SlNCEDI) and SA signalling genes (*SlR1b1, SlPR-P2, SlICS,* and *SlPA*L) in thermotolerant tomato plants [47]. However, studies detailing the molecular mechanisms underpinning Si’s function in controlling hormonal signalling are limited, especially under drought stress [48–51].

Silicon protects photosynthetic machinery and enhances chlorophyll fluorescence/gas exchange parameters such as maximum photochemical efficiency of PSII (Fv/Fm), basal quantum yield (*Fv/Fo*), photochemical quenching (qP), non-photochemical quenching (NPQ), actual photochemical efficiency of PSII (ΦPII), net photosynthesis (Pn), stomatal conductance (gs), intercellular CO_2_ concentration (Ci), transpiration rate (Tr) and photosynthetic enzymes such as RuBP carboxylase and PEP carboxylase [18, 52–55]. Silicon upregulated genes encoding PS I and PSII core proteins (PsbH, PsbB, PsbP, PsbQ, PsbW, Psb28 and PsbD) in heat-stressed wheat plants and drought-stressed tomato plants and maintained photosynthetic electron transport rate (ETR) and photochemical efficiency [56, 57].

Most of the studies mentioned above are conducted in high Si accumulator plants and to the best of our knowledge, no studies are published on Si-mediated drought stress tolerance responses, at the molecular level based on high throughput RNA seq analysis, in a legume plant (a low Si accumulator). Even though the physiological and biochemical mechanisms of Si-mediated drought stress tolerance in lentil plants have been studied recently [7, 8, 19, 54] concerning the molecular response still needs to be investigated. Thus, this study comprehensively explored Si-mediated stress tolerance responses in drought-tolerant and sensitive lentil genotypes using high-throughput RNA-seq analysis and provided novel insight into gene regulation of various pathways involved in Si-mediated drought stress tolerance.

## Results

### Total RNA integrity and cDNA library preparation

**Fig. 1:**
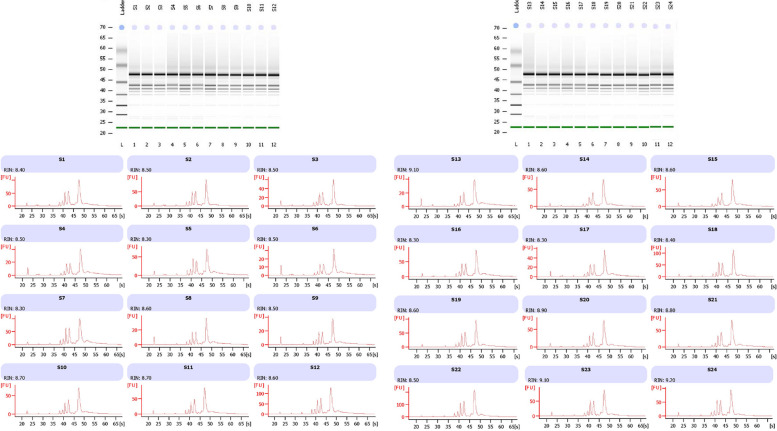
Bioanalyzer output of RNA electrophoresis and corresponding electropherograms with fluorescence unit (FU) on x-axis and time (sec) on Y axis, with peaks of 18S and 28S rRNA along with RNA integrity number (RIN) values

All the RNA samples passed the quality check for library construction and sequencing, with RNA integrity number (RIN) values ranging between 8.3 and 9.2 (Table 1). The concentration of RNA samples (ng/μL) ranged from 273–896 and A260/A280 ratio was greater than 2.1 for all the samples (Fig. 1; Table 1).

**Table 1:** RNA concentration, A260/280 ratio and RNA integrity number (RIN) value of lentil genotypes, ILL 6002 (G1) and ILL 7537 (G2) under different treatments

Classification	Replication	Sample Name	RNA Concentration (ng/µL)	A 260/A280 ratio	RIN value
G1C	R1	S1	597	2.80	8.50
G1C	R2	S2	671	2.20	8.50
G1C	R3	S3	666	2.20	8.50
G1Si	R1	S10	441	2.10	8.70
G1Si	R2	S11	431	2.10	8.70
G1Si	R3	S12	721	2.10	8.60
G1D	R1	S4	273	2.10	8.50
G1D	R2	S5	418	2.10	8.30
G1D	R3	S6	240	2.10	8.50
G1DSi	R1	S7	623	2.20	8.30
G1DSi	R2	S8	301	2.20	8.60
G1DSi	R3	S9	471	2.20	8.50
G2C	R1	S13	323	2.10	9.10
G2C	R2	S14	325	2.10	8.60
G2C	R3	S15	327	2.10	8.60
G2Si	R1	S22	896	2.10	8.50
G2Si	R2	S23	411	2.10	9.10
G2Si	R3	S24	409	2.10	9.20
G2D	R1	S16	382	2.10	8.30
G2D	R2	S17	351	2.10	8.30
G2D	R3	S18	486	2.10	8.40
G2DSi	R1	S19	380	2.20	8.60
G2DSi	R2	S20	337	2.10	8.90
G2DSi	R3	S21	332	2.10	8.80

*Abbreviations* used in this table are *RIN* RNA integrity number and A260/280-Absorbance 260/280, *C* Control, *Si* Silicon alone, *D* Drought stress, *DSi* Drought stress supplemented with Si

### Mapping and identification of differentially expressed genes

RNA samples from the leaves of the two lentil genotypes, grown under different treatments, were used for sequencing by the Illumina Hiseq Analyzer to reveal the role of Si in various molecular regulatory mechanisms to alleviate the adverse effect of drought stress in selected lentil genotypes. Approximately 22 million reads were generated from each sample with an average data yield of 4.52 Gbp (Table 2). Nearly 85% of the pseudo-aligned reads were mapped to the lentil draft reference genome (Table 2).

**Table 2:** Total number of processed and pseudo aligned reads (with percentage) of lentil genotypes, ILL 6002 (G1) and ILL 7537 (G2) under different treatments

Classification	Replication	Sample	Processed Reads	Data yield (Gbp)	Pseudo-aligned Reads	Pseudo-aligned Reads (%)
G1C	R1	S1	21,892,045	4.42	18,208,271	83.17
G1C	R2	S2	21,924,745	4.60	18,000,827	82.10
G1C	R3	S3	21,915,738	5.28	18,775,727	85.67
G1D	R1	S4	21,809,026	4.93	17,765,662	81.46
G1D	R2	S5	21,733,770	5.30	17,927,902	82.48
G1D	R3	S6	23,404,134	2.87	19,325,617	82.57
G1Si	R1	S10	22,782,025	4.52	20,410,088	89.59
G1Si	R2	S11	26,099,944	4.14	21,709,176	83.18
G1Si	R3	S12	24,388,240	4.43	20,520,190	84.14
G1DSi	R1	S7	22,193,811	4.70	19,074,707	85.95
G1DSi	R2	S8	23,970,019	3.93	20,953,844	87.42
G1DSi	R3	S9	26,325,246	4.53	23,039,587	87.52
G2C	R1	S13	26,259,109	4.71	22,132,810	84.29
G2C	R2	S14	14,182,967	5.01	12,378,143	87.27
G2C	R3	S15	23,280,039	3.36	20,091,261	86.30
G2D	R1	S16	19,461,919	4.64	15,860,242	81.49
G2D	R2	S17	22,447,642	4.43	19,269,385	85.84
G2D	R3	S18	22,378,808	4.40	18,916,885	84.53
G2Si	R1	S22	16,606,887	4.85	14,239,226	85.74
G2Si	R2	S23	22,986,096	5.32	19,661,350	85.54
G2DSi	R1	S19	20,494,541	4.39	16,986,853	82.88
G2DSi	R2	S20	23,295,632	4.73	19,684,298	84.50
G2DSi	R3	S21	24,810,152	4.49	20,189,617	81.38
Average	R3		22,375,762.39	4.52	18,918,333.39	84.57

**Fig. 2:**
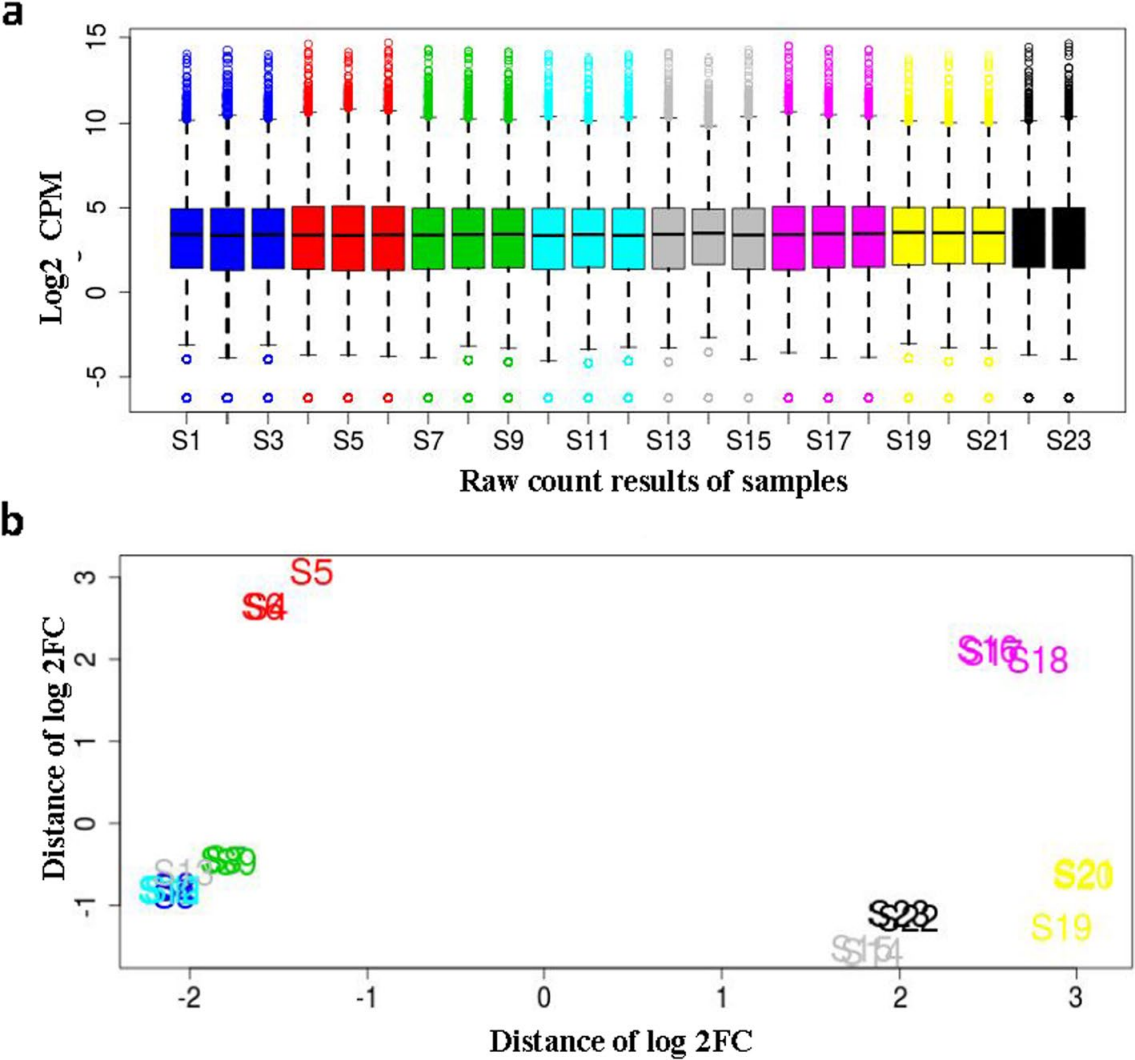
Normalization and **b** multidimensional scaling (MDS) plots of raw count results of all samples from two lentil genotypes under different treatments

A core set of DEGs from the two lentil genotypes, under various treatments, were examined and analysed to identify the key genes involved in Si-induced drought stress tolerance. The distribution of samples (after normalization and the clustering of three replicates from each sampling group) are shown in the normalization plot and multidimensional scaling (MDS) plot (Fig. 2a and b). The biological replicates clustered together for each treatment, suggesting high reliability and accuracy of the RNA sequencing data.

**Fig. 3:**
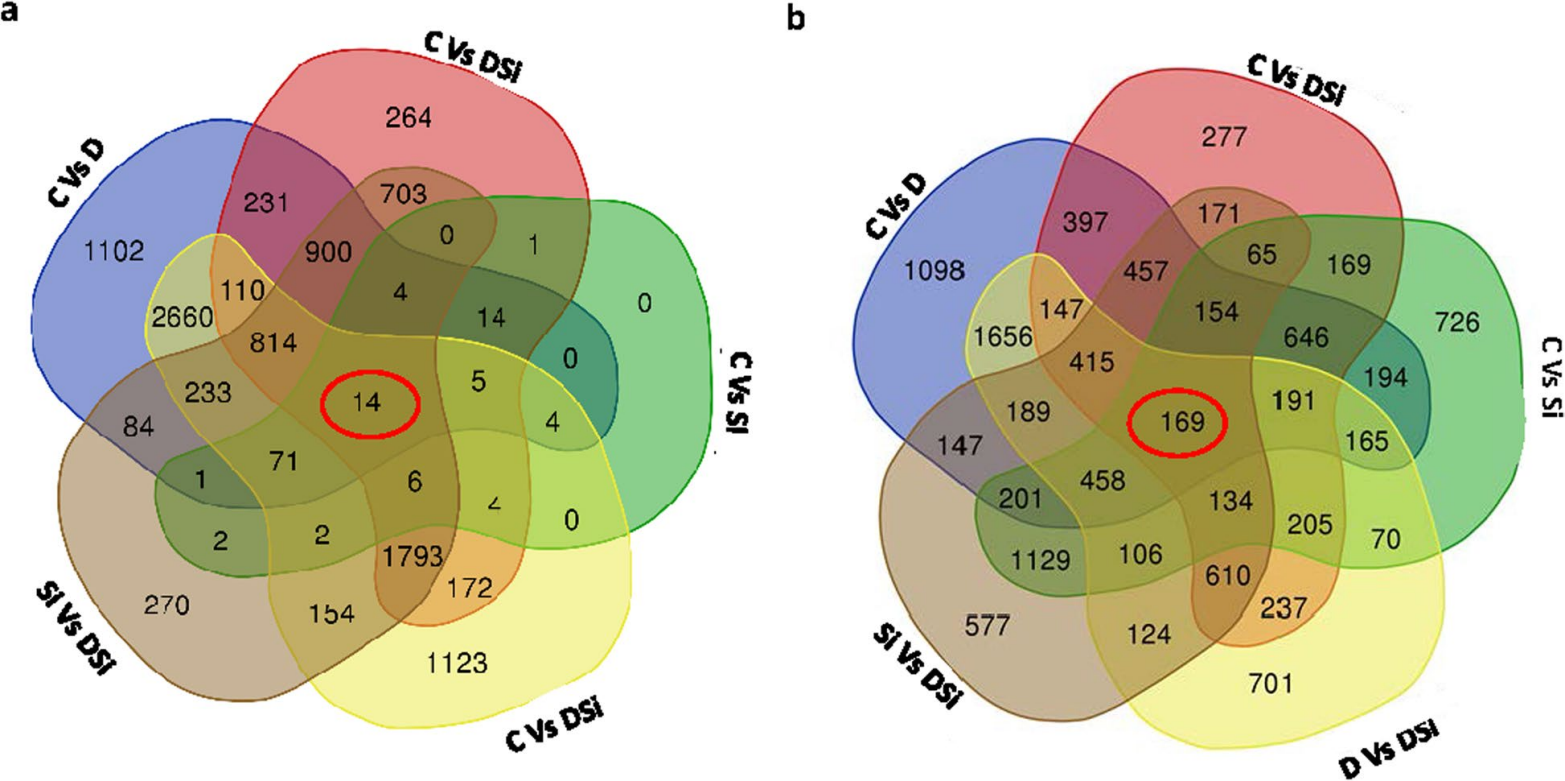
Venn diagram showing genes differently expressed for each comparison and all the possible combinations for (**a**) the drought tolerant genotype (ILL 6002) and (**b**) the drought sensitive genotype (ILL 7537). Abbreviations used include (i) control vs. drought (C vs. D), (ii) control vs. silicon alone (C vs. Si), (iii) drought vs drought stress supplemented with Si (D vs. DSi) (iv) silicon alone vs. drought stress supplemented with Si (Si vs. DSi), (v) control vs. drought stress supplemented with Si (C vs. DSi)

High-throughput RNA-sequencing analysis was performed in the following five combinations from the two genotypes: (i) control vs. drought (C vs. D), (ii) control vs. Si alone (C vs. Si), (iii) drought vs. drought stress supplemented with Si (D vs. DSi) (iv) Si alone vs. drought stress supplemented with Si (Si vs. DSi), and (v) control vs. drought stress supplemented with Si (C vs. DSi). The specific and common DEGs were identified for all the possible combinations, from drought tolerant and sensitive genotypes, as shown in the Venn diagram (Fig. 3a and b). Among the DEGs, 14 unique genes were identified as common to all the comparisons and all the possible combinations for the drought-tolerant genotype (ILL 6002), whereas 169 DEGs were found from the drought-sensitive (ILL 7537) genotype.

**Fig. 4:**
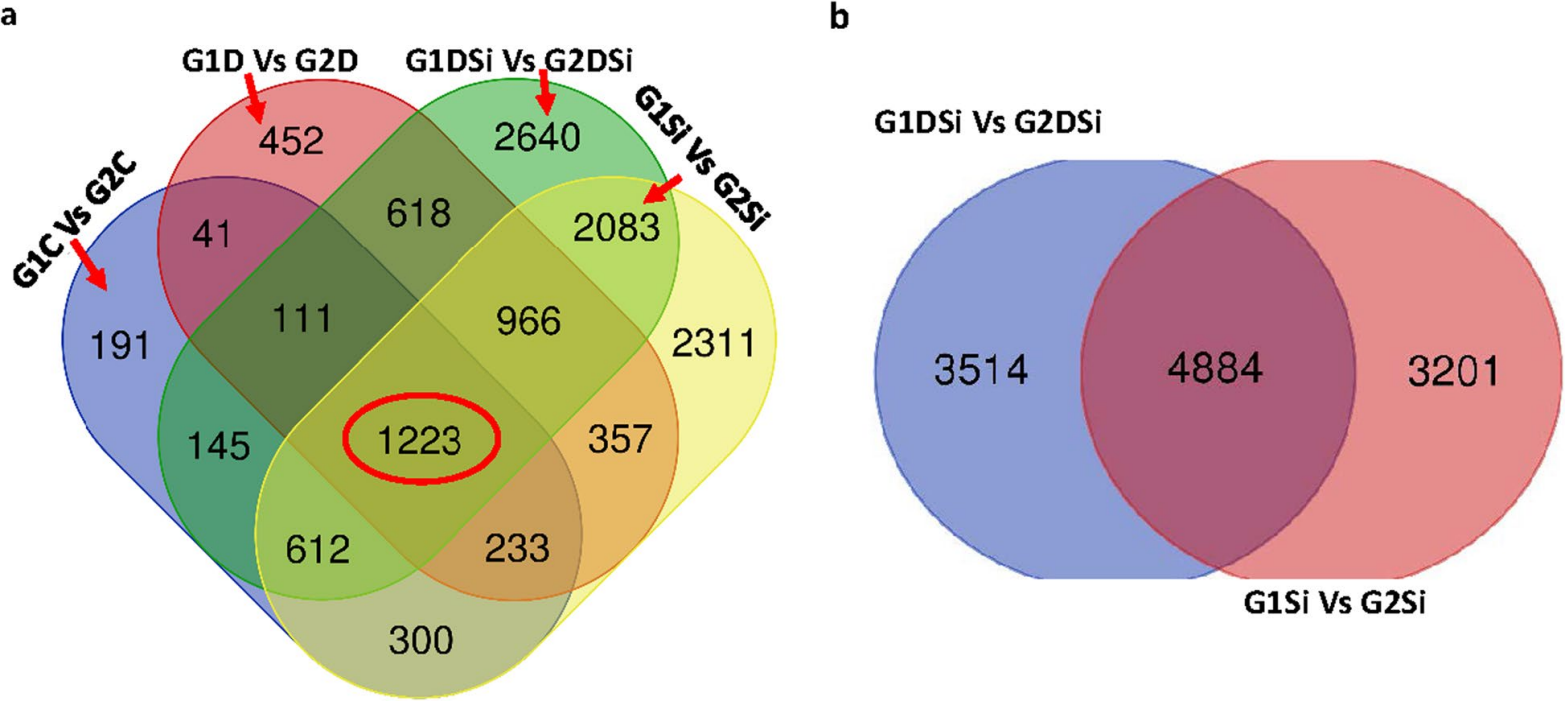
**a** Venn diagram showing DEGs across four comparisons of various treatments in ILL 6002 (G1) and ILL 7537 (G2) under drought stress supplemented with Si and Si alone treatments. Abbreviations used include (i) G1D vs. G2D (G1 drought vs. G2 Drought), (ii) G1C vs. G2C (G1 control vs. G2 control) (iii) G1DSi vs. G2 DSi (G1 drought silicon vs. G2 drought stress supplemented with Si) (iv) G1Si vs. G2Si (G1 silicon alone vs. G2 silicon alone). **b** shows co-expressed and specifically expressed genes in two genotypes. Arrows point to the DEGs exclusively found in each comparison

When the two genotypes were compared for different treatments such as (i) G1 drought vs. G2 drought, (ii) G1 control vs. G2 control (iii) G1DSi vs. G2 DSi and (iv) G1 Si alone vs. G2 Si alone, a total of 1223 genes were differentially expressed (Fig. 4a). Interestingly, when two genotypes were compared for treatments involving Si (G1 Si alone vs. G2 Si alone and G1DSi vs. G2 DSi, 4884 genes were expressed differently (Fig. 4b).

**Fig. 5:**
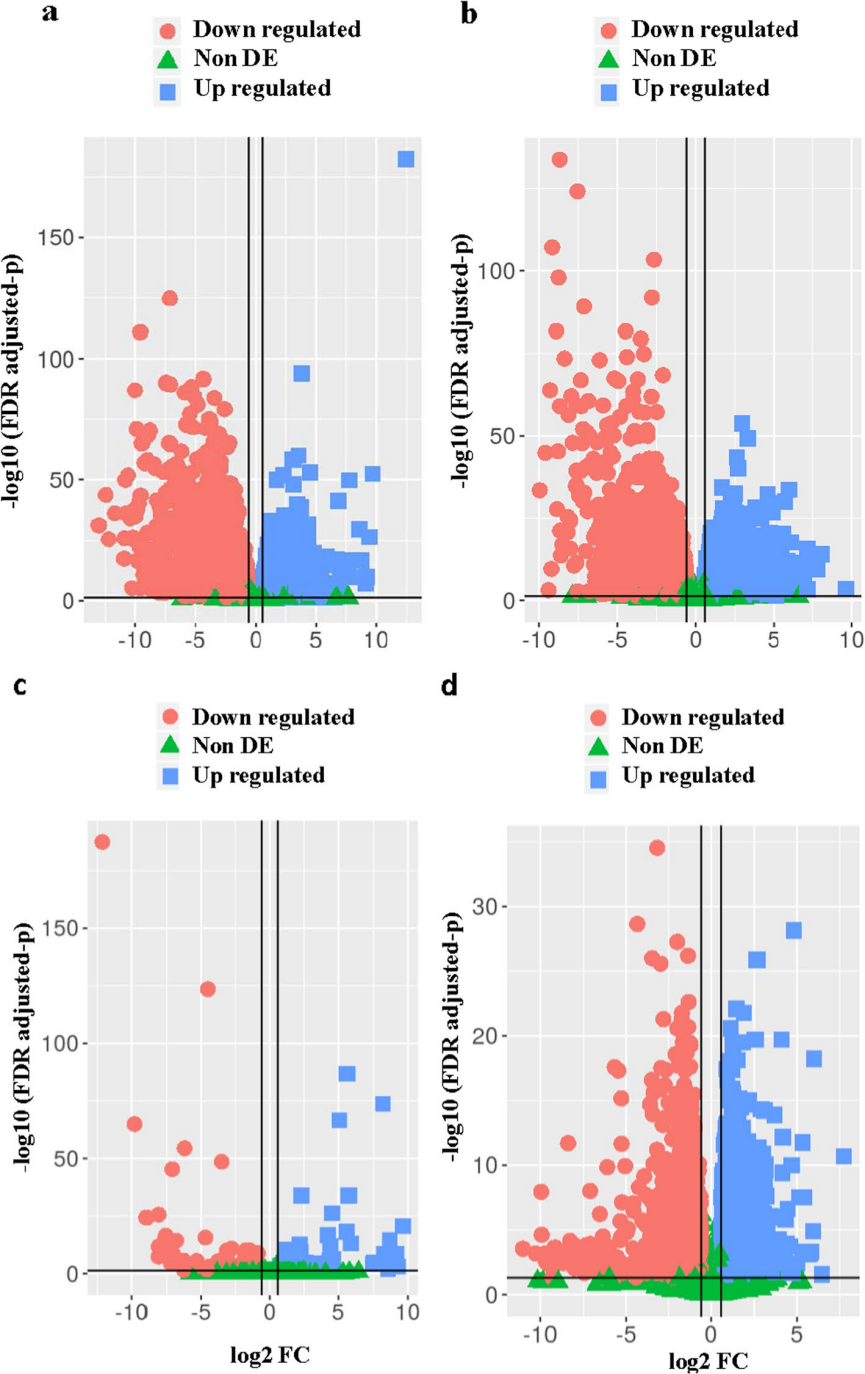
**a-d** The volcano plot of DEGs for drought stress and Si Vs drought treatments of and for control vs. Silicon alone treatments of (**c**) ILL 6002 and (**d**) ILL 7537. The abscissa indicates the level of expression as log2 of the folds change (log 2F) and the ordinates are -log10 (*p*-value). Each symbol represents a gene. The red circle represents the down regulated DEGs, blue squares represent the up regulated DEGs, and green triangle represent non-DEGs. DEG- differentially expressed gene

During drought stress, the drought-sensitive genotype, ILL 7537, showed a slightly higher number of DEGs (6683) than the drought-tolerant genotype, ILL 6002 (6246), when compared with their respective controls (Table 3). Interestingly, different types of genes were expressed in response to drought stress by the two genotypes and more importantly, the drought-sensitive one showed higher DEGs (4781) in the C vs. Si treatments, compared with the 127 DEGs in the drought-tolerant genotype, ILL 6002. However, a total of 7164 genes were differentially expressed in the tolerant genotype, which comprised 3695 upregulated and 3469 downregulated genes, compared with a total of 5576 DEGs in the sensitive genotypes, including 3077 upregulated and 2499 downregulated genes, under D vs. DSi. Overall, Si supplementation under drought stress expressed the specific sets of genes in both the sensitive and tolerant genotypes (Table 3). The volcano plot showed the overall distribution of data points. The significance of the measured differences in expression levels and the selection of upregulated and downregulated DEGs from the two genotypes, under drought stress and Si vs. drought, and control vs. Si alone treatments, is illustrated in Fig. 5a—d. The most exciting finding from this comparison was the higher number of DEGs being detected from ILL 7537 (drought-sensitive) under control vs. Si alone treatments, which suggests that Si interacted with multiple and specific defence pathways/mechanisms in the drought-sensitive genotype to mitigate drought stress. Furthermore, these results indicate that Si supplementation can enhance a drought-sensitive plant’s ability to perform better, even under non-stress conditions.

**Table 3:** The number of up regulated and down regulated differentially expressed genes in drought tolerant (ILL 6002) and sensitive (ILL 7537) lentil genotypes under different treatments

	**Differentially expressed genes in ILL 6002**			**Differentially expressed genes in ILL 7537**		
Comparison types	Up regulated	Down regulated	Total	Up regulated	Down regulated	Total
C vs. D	3056	3190	6246	3733	2950	6683
C vs. Si	52	75	127	1988	2793	4781
D vs. DSi	3695	3469	7164	3077	2499	5576
Si vs. DSi	2350	2700	5050	2145	2960	5105
C vs. DSi	2415	2619	5034	2166	2277	4443
Total genes	11,568	12,053	23,621	13,109	13,479	26,588

*Abbreviations* used in this table are *C* Control, *Si* Silicon alone, D *D*rought stress, *DSi* Drought stress supplemented with Si and Si-Silicon alone

**Fig. 6:**
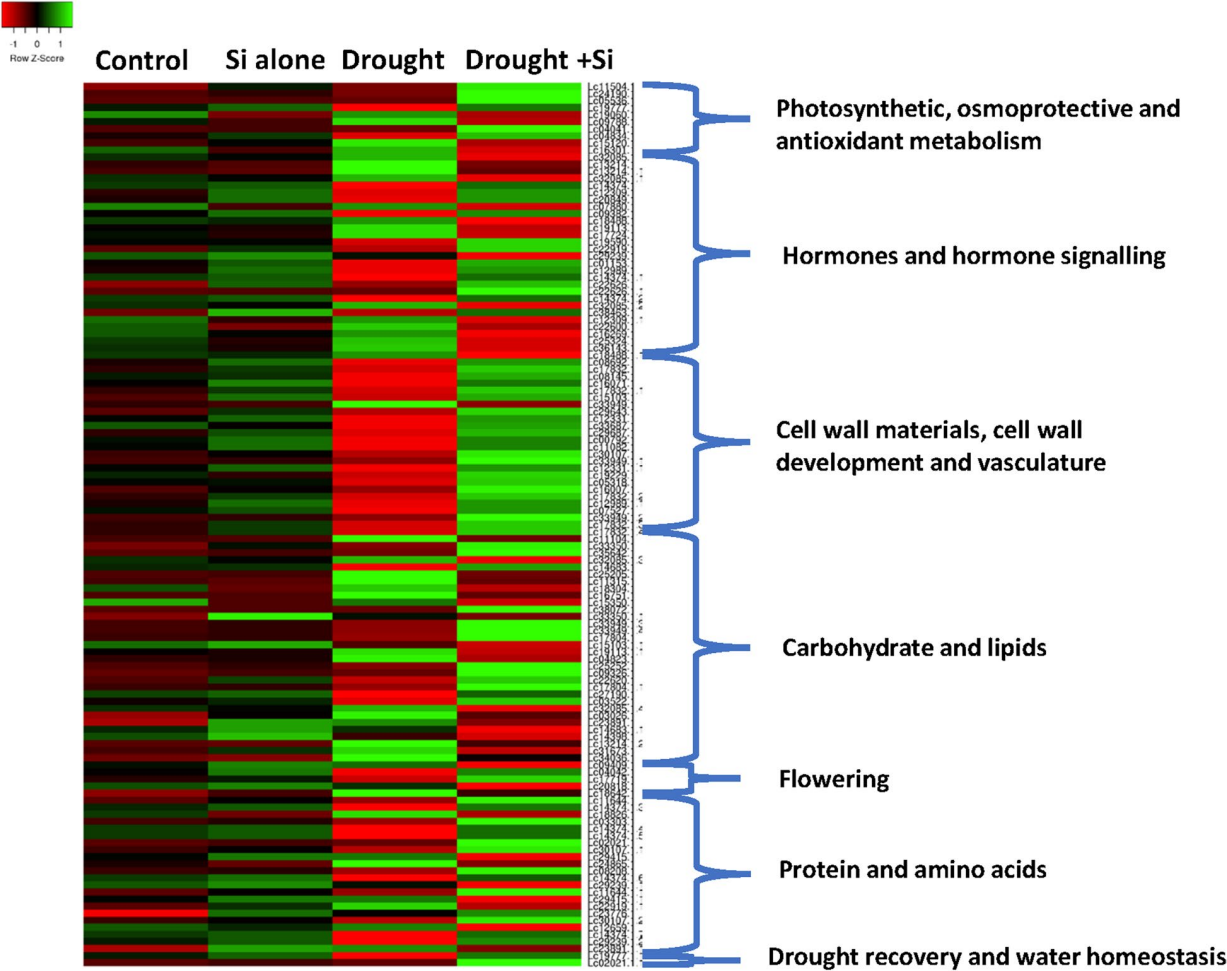
Heatmap representing the expression of differentially expressed candidate genes in response to Si treatment during drought stress in ILL 6002 in DSi vs. D treatment (*p* ≤ 0.05). The colour key represents the normalised log transformed counts. Red indicates low expression, black indicates intermediate expression and green indicates high expression. Each column represents an experimental condition, and each row represents a gene (*p* ≤ 0.05, ≥ 1.5—folds change). The significantly (*P* ≤ 0.05) enriched biological process GO terms are shown on the right side of each cluster. Abbreviations used in this Figure are DSi—Drought stress supplemented with Si and D—Drought stress

**Fig. 7:**
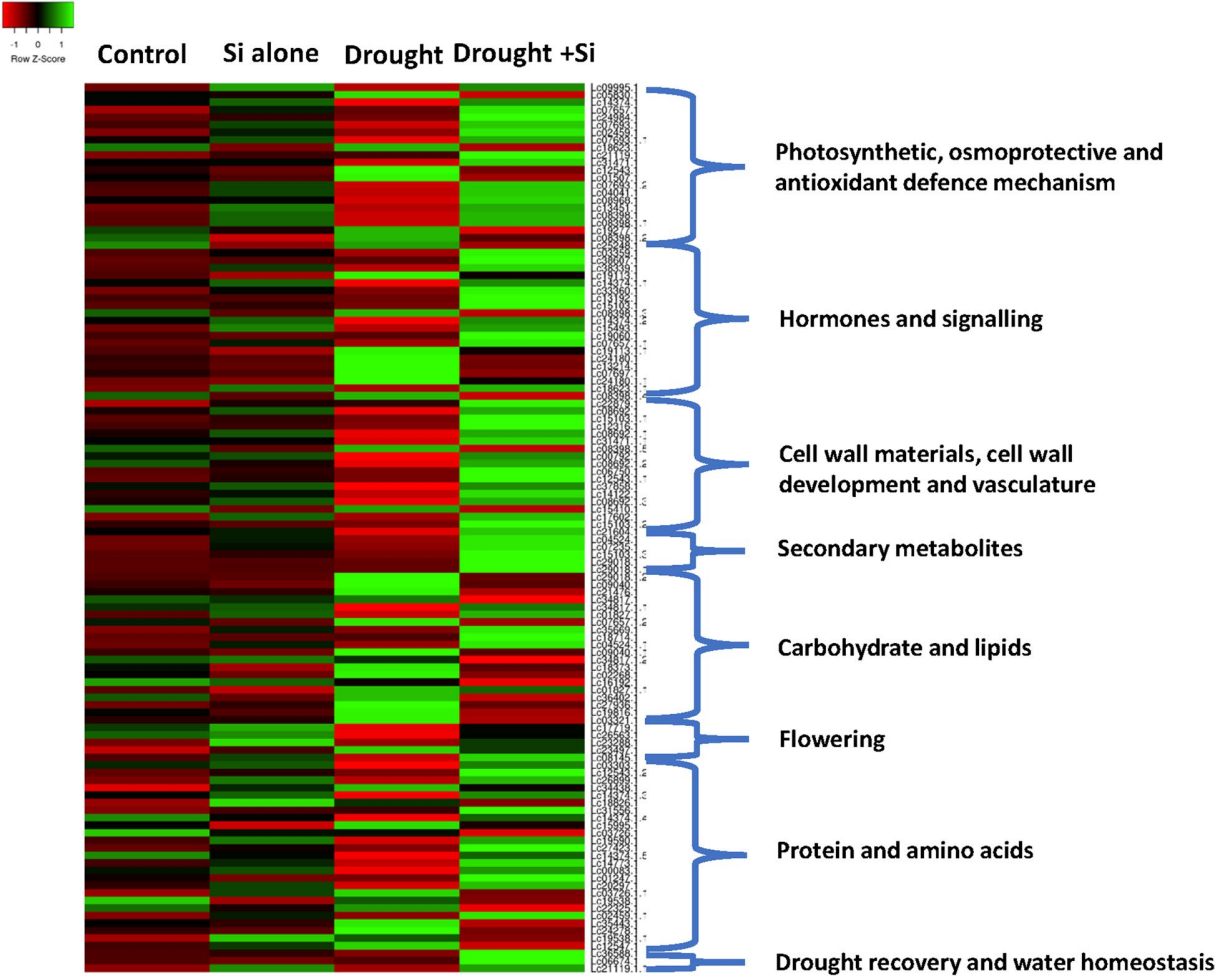
Heatmap representing the expression of differentially expressed candidate genes in response to Si application during drought stress in ILL 7537 in DSi vs. D treatment (*p* ≤ 0.05). The colour key represents the normalised log transformed counts. Red indicates low expression, black indicates intermediate expression and green indicates high expression. Each column represents an experimental condition, and each row represents a gene (*p* ≤ 0.05, ≥ 1.5-folds change). The significantly (*P* ≤ 0.05) enriched biological process GO terms are shown on the right side of each cluster. Abbreviations used in this Figure are DSi—Drought stress supplemented with Si and D—Drought stress

The current study identified the candidate genes behind Si-mediated drought stress tolerance from both the genotypes under drought vs. drought stress with Si treatments, ascertaining the significance (*p* ≤ 05) of the DEGs related to drought tolerance mechanisms. The heatmap shows these selected DEGs' expression patterns (Figs. 6 and 7). Interestingly, the major group of genes among these highly expressed DEGs were related to photosynthetic processes, osmoprotective function, antioxidant metabolisms, hormonal regulation and signalling, cell wall and vasculature biogenesis, carbohydrate and lipid metabolism, protein and amino acid metabolism, secondary metabolite production, flowering, drought recovery and water homeostasis.

### Photosynthetic process, osmoprotective function and antioxidant metabolism related genes expression in response to Si-mediated drought stress tolerance

The DEGs related to photosynthetic processes, such as chlorophyll biosynthesis, qP, relocation of chloroplast, guard cell synthesis, carbon fixation, and plastoquinone biosynthesis, were upregulated. In contrast, the genes controlling movements and openings of stomata were downregulated with Si supplementation under drought stress in both genotypes (Table 4). Upregulated DEGs related to chlorophyll biosynthetic processes were found in both genotypes. DEGs responsible for osmotic stress response were upregulated with the downregulation of proline biosynthesis and metabolism genes in both genotypes under drought stress with Si supplementation. More DEGs were positively regulated in response to oxidative stress and antioxidant metabolism in drought-sensitive genotype, ILL 7537, compared to the tolerant one (ILL 6002). Moreover, genes related to defence responses and detoxification of cellular oxidants were upregulated and differentially expressed in the drought-sensitive genotype with Si supplementation during drought stress, compared with drought stress without Si supplementation.

**Table 4:** Differentially expressed genes related to photosynthetic, osmoprotective and antioxidant metabolism in lentil genotypes for DSi vs. D comparison

Gene	Description	GO ID	Up/Down- regulated (U/D)
**ILL 6002**			
Lc04041.1	chlorophyll biosynthetic process	GO:0015995	U
Lc11504.1	chlorophyll metabolic process	GO:0015994	U
Lc24190.1	chloroplast relocation	GO:0009902	U
Lc05536.1	plastoquinone biosynthetic process	GO:0010236	U
Lc04834.1	guard cell morphogenesis	GO:0010442	U
Lc19777.1	response to osmotic stress	GO:0006970	U
Lc15120.1	regulation of stomatal opening	GO:1,902,456	D
Lc16301.1	stomatal movement	GO:0010118	D
Lc19060.1	proline biosynthetic process	GO:0006561	D
Lc09788.1	hydrogen peroxide biosynthetic process	GO:0050665	D
**ILL 7537**			
Lc09995.1	chlorophyll biosynthetic process	GO:0015995	U
Lc07693.1	photosynthesis	GO:0015979	U
Lc04041.1	chlorophyll biosynthetic process	GO:0015995	U
Lc08968.1	photosynthetic electron transport in PSI	GO:0009773	U
Lc13451.1	nonphotochemical quenching	GO:0010196	U
Lc07693.1	carbon fixation	GO:0015977	U
Lc21119.1	oxidation–reduction process	GO:0055114	U
Lc31471.1	regulation of membrane potential	GO:0042391	U
Lc07657.1	response to osmotic stress	GO:0006970	U
Lc21119.1	oxidation–reduction process	GO:0055114	U
Lc02459.1	response to oxidative stress	GO:0006979	U
Lc08398.1	positive regulation of response to oxidative stress	GO:1,902,884	U
Lc08398.1	cellular oxidant detoxification	GO:0098869	U
Lc24984.1	glutathione metabolic process	GO:0006749	U
Lc14374.1	defence response	GO:0006952	U
Lc05830.1	regulation of stomatal movement	GO:0010119	D
Lc01507.1	regulation of chlorophyll catabolic process	GO:0010271	D
Lc19277.1	PSII associated light-harvesting complex II catabolic process	GO:0010304	D
Lc05830.1	regulation of stomatal movement	GO:0010119	D
Lc25248.1	chloroplast organization	GO:0009658	D
Lc09995.1	regulation of stomatal opening	GO:1,902,456	D
Lc18623.1	proline metabolic process	GO:0006560	D
Lc08398.1	superoxide metabolic process	GO:0006801	D
Lc12543.1	hydrogen peroxide metabolic process	GO:0042743	D
Lc08398.1	superoxide metabolic process	GO:0006801	D
Lc12543.1	hydrogen peroxide metabolic process	GO:0042743	D

*Abbreviations* used in this table are *DSi* Drought stress supplemented with Si and *D* Drought stress

### Role of important phytohormones in S-induced drought tolerance in lentil

In the present study, different expression levels of genes related to hormone and hormone signalling pathways were observed in both tolerant and sensitive genotypes (Table 5). Upregulated DEGs were identified for AUX, karrikin (KAR) and polyamines (PA), while downregulated DEGs were found for ET and JA metabolism in both the genotypes, in response to Si supplementation under drought stress. Furthermore, the drought-sensitive genotype showed upregulated DEGs for BR and downregulated DEGs for CK which were not identified from the drought-tolerant genotypes. Upregulated DEGs for negative regulation of the GA-mediated signalling pathway were identified in the drought-tolerant genotype. Interestingly, while the drought-tolerant genotype showed the downregulation of the expression of genes involved in SA biosynthesis, drought sensitive-genotype showed the reverse. Additionally, the gene related to AUX biosynthetic processes was found to be common for both genotypes.

**Table 5:** Differentially expressed genes related to hormones and hormones signalling in lentil genotypes for DSi vs. D comparison

Gene	Description	GO ID	Up/Down regulated (U/D)
**ILL 6002**			
Lc20849.1	response to karrikin	GO:0080167	U
Lc14374.1	auxin metabolic process	GO:0090354	U
Lc09382.1	protein localization involved in auxin Polar transport	GO:1,901,703	U
Lc14374.1	cellular response to auxin stimulus	GO:0071365	U
Lc14374.1	auxin biosynthetic process	GO:0009851	U
Lc09382.1	protein localization involved in auxin Polar transport	GO:1,901,703	U
Lc22626.1	S-adenosyl methioninamine biosynthetic process	GO:0006557	U
Lc22626.1	adenosylmethionine decarboxylase activity	GO:0004014	U
Lc38463.1	polyamine metabolic process	GO:0006595	U
Lc22919.1	signalling	GO:0023052	U
Lc01153.1	signalling receptor activity	GO:0038023	U
Lc12989.1	negative regulation of gibberellic acid mediated signalling pathway	GO:0009938	U
Lc19113.1	auxin binding	GO:0010011	D
Lc36143.1	ethylene biosynthetic process	GO:0009693	D
Lc25324.1	ethylene binding	GO:0051740	D
Lc17724.1	( +)-abscisic acid 8'-hydroxylase activity	GO:0010295	D
Lc18488.1	salicylic acid metabolic process	GO:0009696	D
Lc32085.1	response to jasmonic acid	GO:0009753	D
Lc07880.1	regulation of jasmonic acid mediated signalling pathway	GO:2,000,022	D
Lc32085.1	jasmonic acid biosynthetic process	GO:0009695	D
Lc13214.1	allene-oxide cyclase activity	GO:0046423	D
Lc24180.1	allene oxide synthase activity	GO:0009978	D
Lc17724.1	( +)-abscisic acid 8'-hydroxylase activity	GO:0010295	D
Lc18488.1	salicylic acid metabolic process	GO:0009696	D
Lc12309.1	hormone biosynthetic process	GO:0042446	D
Lc32085.1	hormone biosynthetic process	GO:0042446	D
Lc12309.1	positive regulation of gibberellin biosynthesis	GO:0010372	D
Lc22600.1	gibberellic acid homeostasis	GO:0010336	D
Lc16269.1	hormone-mediated signalling pathway	GO:0009755	D
Lc12309.1	positive regulation of gibberellin biosynthesis	GO:0010372	D
Lc22600.1	gibberellic acid homeostasis	GO:0010336	D
**ILL 7537**			
Lc03359.1	response to karrikin	GO:0080167	U
Lc14374.1	auxin biosynthetic process	GO:0009851	U
Lc15493.1	indoleacetic acid metabolic process	GO:0009683	U
Lc14374.1	auxin metabolic process	GO:0009850	U
Lc18623.1	polyamine metabolic process	GO:0006595	U
Lc19060.1	regulation of abscisic acid biosynthetic process	GO:0010115	U
Lc38607.1	regulation of brassinosteroid biosynthetic process	GO:0010422	U
Lc38339.1	regulation of salicylic acid biosynthetic process	GO:0080142	U
Lc33360.1	hormone catabolic process	GO:0042447	U
Lc13192.1	cellular hormone metabolic process	GO:0034754	U
Lc15103.1	signal transduction	GO:0007165	U
Lc07657.1	response to cytokinin	GO:0009735	D
Lc07697.1	regulation of jasmonic acid mediated signalling pathway	GO:2,000,022	D
Lc24180.1	jasmonic acid metabolic process	GO:0009694	D
Lc08398.1	negative regulation of abscisic acid-activated signalling pathway	GO:0009788	D
Lc24180.1	allene-oxide cyclase activity	GO:0046423	D
Lc13214.1	allene oxide synthase activity	GO:0046423	D
Lc08398.1	negative regulation of signalling	GO:0023057	D

*Abbreviations* used in this table are *DSi *Drought stress supplemented with Si, *D* Drought stress

### Differentially regulated genes involved in cell wall development, synthesis of cell wall materials and vasculature biogenesis

In this study, Si supplementation of drought stressed lentil genotypes resulted in the upregulation of DEGs related to primary and secondary cell wall biogenesis and organization (Table 6). Furthermore, Si upregulated the genes responsible for synthesising and metabolising cell wall material such as cellulose, lignin, xyloglucan, chitin and xylan. Genes regulating phenylpropanoid pathway, a source of lignin formation in plant cells and lignin metabolic processes, were also upregulated, suggesting Si’s role in physical defence mechanisms in lentils under drought stress. Upregulated DEGs were identified for the histogenesis, development and patterning of the vascular tissues (xylem and phloem) and phloem transport in both genotypes, in response to Si under drought stress.

**Table 6:** Differentially expressed genes related to the synthesis and development of cell wall materials and vasculature genes in lentil genotypes for DSi vs. D comparison

Gene	Description	GO ID	Up/Down regulated (U/D)
**ILL 6002**			
Lc08692.1	plant-type secondary cell wall biogenesis	GO:0009834	U
Lc17832.1	plant-type primary cell wall biogenesis	GO:0009833	U
Lc33949.1	cell wall organization or biogenesis	GO:0071554	U
Lc17832.1	cell wall biogenesis	GO:0042546	U
Lc16071.1	xylan biosynthetic process	GO:0045492	U
Lc17832.1	cellulose metabolic process	GO:0030243	U
Lc23776.1	phenylpropanoid biosynthetic process	GO:0009699	U
Lc15103.1	lignin metabolic process	GO:0009808	U
Lc33687.1	response to chitin	GO:0010200	U
Lc00792.1	vasculature development	GO:0001944	U
Lc11082.1	xylem and phloem pattern formation	GO:0010051	U
Lc30107.1	phloem transport	GO:0010233	U
Lc33949.1	cell wall organization	GO:0071555	U
Lc19229.1	phloem or xylem histogenesis	GO:0010087	U
Lc05318.1	cellulose microfibril organization	GO:0010215	U
Lc16007.1	meristem structural organization	GO:0009933	U
Lc17832.1	cell division	GO:0051301	U
Lc12989.1	meiotic cytokinesis	GO:0033206	U
Lc07527.1	microtubule cytoskeleton organization involved in mitosis	GO:1,902,850	U
Lc29643.1	microtubule-based movement	GO:0007018	U
Lc12331.1	phragmoplast assembly	GO:0000914	U
Lc08145.1	cell communication	GO:0007154	U
Lc29687.1	extracellular matrix organization	GO:0030198	U
Lc33949.1	defence response by callose deposition	GO:0052542	D
**ILL 7537**			
Lc08692.1	plant-type secondary cell wall biogenesis	GO:0009834	U
Lc08692.1	plant-type primary cell wall biogenesis	GO:0009833	U
Lc12316.1	vasculature development	GO:0001944	U
Lc00792.1	primary meristem tissue development	GO:0010065	U
Lc06750.1	phloem transport	GO:0010233	U
Lc12543.1	xylem development	GO:0010089	U
Lc37858.1	xylem and phloem pattern formation	GO:0010051	U
Lc37858.1	regulation of cell wall organization	GO:1,903,338	U
Lc14122.1	xyloglucan metabolic process	GO:0010411	U
Lc08692.1	cellulose biosynthetic process	GO:0030244	U
Lc22879.1	response to chitin	GO:0010200	U
Lc12543.1	regulation of phenylpropanoid metabolic processes	GO:2,000,762	U
Lc15103.1	lignin metabolic process	GO:0009808	U
Lc08398.1	negative regulation of cell communication	GO:0010648	D
Lc15410.1	suberin biosynthetic process	GO:0010345	D

*Abbreviations* used in this table are *DSi *Drought stress supplemented with Si and *D* Drought stress

### Differentially regulated genes encoding carbohydrate and lipid metabolism

The current study showed an upregulation in carbohydrate metabolism with higher upregulated DEGs in both genotypes under drought stress in response to Si (Table 7). Among the upregulated DEGs, the most important ones were related to the metabolism of starch and trehalose (synthesized especially during freezing and drought stress in plants) [58]. Additionally, downregulated DEGs were found for sucrose metabolism. Downregulated DEGs involved in the metabolism of phospholipids, galactolipids, fatty acids and fatty acid beta-oxidation were identified in both the genotypes under drought stress in response to Si. In addition, downregulated DEGs were also identified for the activity of major enzymes involved in lipid metabolism, such as omega**-**3 fatty acid desaturase, acyl-CoA dehydrogenase and acetyl-CoA C-acyltransferase, which further supports the downregulation of lipid metabolism.

**Table 7:** Differentially expressed genes related to carbohydrate and lipid metabolism in lentil genotypes for DSi vs. D comparison

Gene	Description	GO ID	Up/Down regulated (U/D)
**ILL 6002**			
Lc35422.1	carbohydrate binding	GO:0030246	U
Lc33350.1	starch biosynthetic process	GO:0019252	U
Lc14683.1	glycogen metabolic process	GO:0005977	U
Lc33949.1	glucosyltransferase activity	GO:0046527	U
Lc33949.1	UDP-glycosyltransferase activity	GO:0008194	U
Lc17804.1	trehalose-phosphatase activity	GO:0004805	U
Lc15103.1	O-acyltransferase activity	GO:0008374	U
Lc33949.1	glucosyltransferase activity	GO:0046527	U
Lc33949.1	UDP-glycosyltransferase activity	GO:0008194	U
Lc09326.1	amylopectin biosynthetic process	GO:0010021	U
Lc22620.1	inositol phosphate dephosphorylation	GO:0046855	U
Lc17804.1	trehalose metabolic process	GO:0005991	U
Lc27190.1	glycerol-3-phosphate catabolic process	GO:0046168	U
Lc09326.1	amylopectin biosynthetic process	GO:0010021	U
Lc22620.1	inositol phosphate dephosphorylation	GO:0046855	U
Lc17804.1	trehalose metabolic process	GO:0005991	U
Lc27190.1	glycerol-3-phosphate catabolic process	GO:0046168	U
Lc04042.1	regulation of polysaccharide metabolic p…	GO:0032881	U
Lc17804.1	trehalose-phosphatase activity	GO:0004805	U
Lc37031.1	isoamylase activity	GO:0019156	U
Lc36923.1	esculetin 4-O-beta-glucosyltransferase activity	GO:0102361	U
Lc27347.1	daphnetin 4-O-beta-glucosyltransferase activity	GO:0102359	U
Lc35607.1	glycolate oxidase activity	GO:0008891	U
Lc27190.1	glycerol-3-phosphate dehydrogenase activity	GO:0004367	U
Lc08575.1	glycerol transmembrane transporter activity	GO:0015168	U
Lc17549.1	oligopeptide transmembrane transporter activity	GO:0035673	U
Lc03522.1	phosphatidylinositol dephosphorylation	GO:0046856	U
Lc18304.1	S-glycoside metabolic process	GO:0016143	D
Lc38072.1	oligosaccharide catabolic process	GO:0009313	D
Lc33350.1	galactose metabolic process	GO:0006012	D
Lc19113.1	inositol hexakisphosphate binding	GO:0000822	D
Lc04823.1	galactinol-sucrose galactosyltransferase activity	GO:0047274	D
Lc25252.1	inositol 3-alpha-galactosyltransferase activity	GO:0047216	D
Lc18304.1	S-glycoside metabolic process	GO:0016143	D
Lc38072.1	oligosaccharide catabolic process	GO:0009313	D
Lc33350.1	galactose metabolic process	GO:0006012	D
Lc19113.1	inositol hexakisphosphate binding	GO:0000822	D
Lc03026.1	sucrose metabolic process	GO:0005985	D
Lc23891.1	glucose mediated signalling pathway	GO:0010255	D
Lc14683.1	glycogen catabolic process	GO:0005980	D
Lc09409.1	response to monosaccharide	GO:0034284	D
Lc03026.1	sucrose metabolic process	GO:0005985	D
Lc23891.1	glucose mediated signalling pathway	GO:0010255	D
Lc31673.1	triglyceride metabolic process	GO:0006641	D
Lc34036.1	polyol metabolic process	GO:0019751	D
Lc31673.1	triglyceride metabolic process	GO:0006641	D
Lc04823.1	galactinol-sucrose galactosyltransferase activity	GO:0047274	D
Lc03398.1	inositol 3-alpha-galactosyltransferase activity	GO:0047216	D
Lc11104.1	response to lipid	GO:0033993	D
Lc32085.1	oxylipin biosynthetic process	GO:0031408	D
Lc11315.1	polyol metabolic process	GO:0019751	D
Lc15350.1	fatty acid beta-oxidation	GO:0006635	D
Lc04823.1	galactinol-sucrose galactosyltransferase activity	GO:0047274	D
Lc03398.1	inositol 3-alpha-galactosyltransferase activity	GO:0047216	D
Lc11104.1	response to lipid	GO:0033993	D
Lc32085.1	oxylipin biosynthetic process	GO:0031408	D
Lc11315.1	polyol metabolic process	GO:0019751	D
Lc32085.1	lipid oxidation	GO:0034440	D
Lc29256.1	acyl-CoA dehydrogenase activity	GO:0003995	D
Lc01773.1	acetyl-CoA C-acyltransferase activity	GO:0003988	D
Lc32085.1	lipid oxidation	GO:0034440	D
**ILL 7537**			
Lc29018.1	indole glucosinolate metabolic process	GO:0042343	U
Lc34817.1	fructose 6-phosphate metabolic process	GO:0006002	U
Lc01827.1	trehalose metabolic process	GO:0005991	U
Lc18714.1	glucose import	GO:0046323	U
Lc34817.1	fructose 6-phosphate metabolic process	GO:0006002	U
Lc01827.1	trehalose metabolic process	GO:0005991	U
Lc01827.1	trehalose metabolism in response to stress	GO:0070413	U
Lc18373.1	malate transmembrane transporter activity	GO:0015140	U
Lc01827.1	trehalose-phosphatase activity	GO:0004805	U
Lc04524.1	negative regulation of lipid biosynthetic activity	GO:0051055	U
Lc29018.1	S-glycoside metabolic process	GO:0016143	D
Lc34817.1	disaccharide biosynthetic process	GO:0046351	D
Lc09040.1	phosphatidylinositol phosphorylation	GO:0046854	D
Lc34817.1	sucrose metabolic process	GO:0005985	D
Lc18373.1	malate transport	GO:0015743	D
Lc16192.1	monosaccharide biosynthetic process	GO:0046364	D
Lc27936.1	oligosaccharide catabolic process	GO:0009313	D
Lc19816.1	galactose metabolic process	GO:0006012	D
Lc03321.1	oxylipin biosynthetic process	GO:0031408	D
Lc21476.1	sterol biosynthetic process	GO:0016126	D
Lc34547.1	inositol 3-alpha-galactosyltransferase activity	GO:0047216	D
Lc18175.1	galactinol-sucrose galactosyltransferase activity	GO:0047274	D
Lc19113.1	inositol hexakisphosphate binding	GO:0000822	D
Lc07657.1	response to lipid	GO:0033993	D
Lc35669.1	phospholipid catabolic process	GO:0009395	D
Lc02268.1	galactolipid metabolic process	GO:0019374	D
Lc36402.1	unsaturated fatty acid metabolic process	GO:0033559	D
Lc35669.1	phospholipid catabolic process	GO:0009395	D
Lc15857.1	omega-3 fatty acid desaturase activity	GO:0042389	D

*Abbreviation* used in this table are *DSi *Drought stress supplemented with Si and *D *Drought stress

### Differentially expressed genes involved in protein and amino acid metabolism

Protein and amino acid metabolism-related DEGs were differentially expressed in the studied genotypes under Si-mediated drought stress tolerance, with more upregulated DEGs for protein phosphorylation, protein kinase activity and positive regulation of amino acids (Table 8). Among DEGs for amino acids, DEGs related to phenylalanine and tryptophan were upregulated and those for leucine were downregulated, in both genotypes. Intriguingly, DEGs involved in arginine, asparagine, glutamate and threonine were downregulated in the drought-sensitive genotype under drought stress.

**Table 8:** Differentially expressed genes related to protein and amino acid metabolism in lentil genotypes for DSi vs. D comparison

Gene	Description	GO ID	Up/Down regulated (U/D)
**ILL 6002**			
Lc11644.1	protein phosphorylation	GO:0006468	U
Lc14374.1	positive regulation of cellular amino acid metabolic process	GO:0045764	U
Lc03303.1	amino acid transport	GO:0006865	U
Lc14374.1	tryptophan biosynthetic process	GO:0000162	U
Lc14374.1	regulation of tryptophan metabolic process	GO:0090357	U
Lc02021.1	L-phenylalanine catabolic process	GO:0006559	U
Lc30107.1	oligopeptide transport	GO:0006857	U
Lc14374.1	positive regulation of cellular amino acid metabolic processes	GO:0045764	U
Lc29239.1	DNA-binding transcription factor activity	GO:0003700	U
Lc11644.1	kinase activity	GO:0016301	U
Lc14374.1	regulation of tryptophan metabolic process	GO:0090357	U
Lc23891.1	regulation of ribosome biogenesis	GO:0090069	U
Lc19590.1	transmembrane receptor protein tyrosine kinase signalling pathway	GO:0007169	U
Lc18826.1	leucine catabolic process	GO:0006552	D
Lc29415.1	regulation of ubiquitin protein ligase activity	GO:1,904,666	D
Lc24865.1	protein ubiquitination	GO:0016567	D
Lc08208.1	cellular response to topologically incorrect protein	GO:0035967	D
Lc29415.1	ubiquitin-protein transferase activator activity	GO:0097027	D
Lc22919.1	ubiquitin-protein transferase activity	GO:0004842	D
Lc12659.1	positive regulation of ubiquitin protein ligase activity	GO:1,904,668	D
**ILL 7537**			
Lc08145.1	protein phosphorylation	GO:0006468	U
Lc03303.1	amino acid transport	GO:0006865	U
Lc26899.1	oligopeptide transport	GO:0006857	U
Lc14374.1	tryptophan biosynthetic process	GO:0000162	U
Lc31556.1	positive regulation of MAP kinase activity	GO:0043406	U
Lc14374.1	positive regulation of cellular amino acid metabolic processes	GO:0045764	U
Lc15995.1	cysteine biosynthetic process from serine	GO:0006535	U
Lc19590.1	transmembrane receptor protein tyrosine kinase signalling pathway	GO:0007169	U
Lc27423.1	lysine biosynthetic process	GO:0009085	U
Lc14374.1	regulation of tryptophan metabolic processes	GO:0090357	U
Lc14773.1	amino acid homeostasis	GO:0080144	U
Lc00083.1	L-phenylalanine metabolic process	GO:0006558	U
Lc20297.1	beta-alanine biosynthetic process	GO:0019483	U
Lc02459.1	cysteine biosynthetic process	GO:0019344	U
Lc18826.1	leucine catabolic process	GO:0006552	D
Lc34438.1	regulation of protein import into nucleus	GO:0042306	D
Lc03726.1	protein glutathionylation	GO:0010731	D
Lc01247.1	protein N-linked glycosylation via asparagine	GO:0018279	D
Lc19538.1	arginine catabolic process	GO:0006527	D
Lc22325.1	asparagine biosynthetic process	GO:0006529	D
Lc35443.1	L-glutamate biosynthetic process	GO:0097054	D
Lc24278.1	peptidyl-threonine phosphorylation	GO:0018107	D

*Abbreviations* used in this table are *DSi* Drought stress supplemented with Si and *D* Drought stress

### Differentially regulated genes involved in the production of secondary metabolites in drought sensitive genotype (ILL 7537)

It was somewhat surprising that no DEGs related to the production of secondary metabolites were identified in the drought-tolerant genotype (ILL 6002), in response to Si under drought stress (Table 9). However, upregulated DEGs were found for the biosynthesis and metabolising secondary metabolites, such as alkaloids and flavonoids, in the drought-sensitive genotype (ILL7537). Upregulated DEGs were also identified for isopentenyl diphosphate, the precursor of isoprenoid, involved in the biosynthesis of terpenes and terpenoids. Silicon triggers vital secondary metabolite biosynthetic pathways in plants, especially under stress conditions [59]. The results suggest that Si may also act as a regulatory molecule under drought stress to protect the plant by effectively synthesizing secondary metabolites in drought-sensitive genotypes. It also indicates the existence of genetic differences among different plant genotypes and species for the regulatory mechanism related to secondary metabolite synthesis and their role in stress tolerance.

**Table 9:** Differentially expressed genes related to secondary metabolite metabolism in drought sensitive lentil genotype (ILL 7537) for DSi vs. D comparison

Gene	Description	GO ID	Up/Down regulated (U/D)
Lc17602.1	camalexin biosynthetic process	GO:0010120	U
Lc15103.1	secondary metabolite biosynthetic processes	GO:0044550	U
Lc21604.1	isopentenyl diphosphate biosynthetic process	GO:0019287	U
Lc04524.1	negative regulation of isoprenoid metabolism	GO:0045827	U
Lc07235.1	alkaloid metabolic process	GO:0009820	U
Lc15103.1	flavonoid metabolic process	GO:0009812	U
Lc29018.1	secondary metabolic process	GO:0019748	U

*Abbreviations* used in this table are *DSi* Drought stress supplemented with Si and *D* Drought stress

### Gene expression related to drought recovery and defence response

The most important finding from this study, which confirmed the role of Si as a ‘drought stress alleviator’ in lentils, was the upregulation of genes related to drought tolerance recovery in both the genotypes under Si-mediated drought tolerance (Table 10). The relatively low expression of these DEGs in the control and the drought stress treatments (Table 10) and their high expression in drought stress in response to Si strongly confirm the positive role of Si in drought stress tolerance of lentil genotypes. Additional upregulated genes related to water homeostasis and defence responses, found in drought-sensitive genotype, further underlines the essentiality and potential of Si supplementation to drought-sensitive genotypes under drought stress.

**Table 10:** Differentially expressed genes related to drought recovery in lentil genotypes for DSi vs. D comparison

Gene	Description	GO ID	Up/Down regulated (U/D)
**ILL 6002**			
Lc19777.1	response to water	GO 0009415	U
Lc02021.1	drought recovery	GO:0009819	U
**ILL 7537**			
Lc36588.1	water homeostasis	GO:0030104	U
Lc06674.1	drought recovery	GO:0009819	U
Lc21119.1	defence response	GO:0006952	U

*Abbreviations* used in this table are *DSi* Drought stress supplemented with Si and *D* Drought stress

### Gene ontology (GO) annotation of differentially expressed genes

**Fig. 8:**
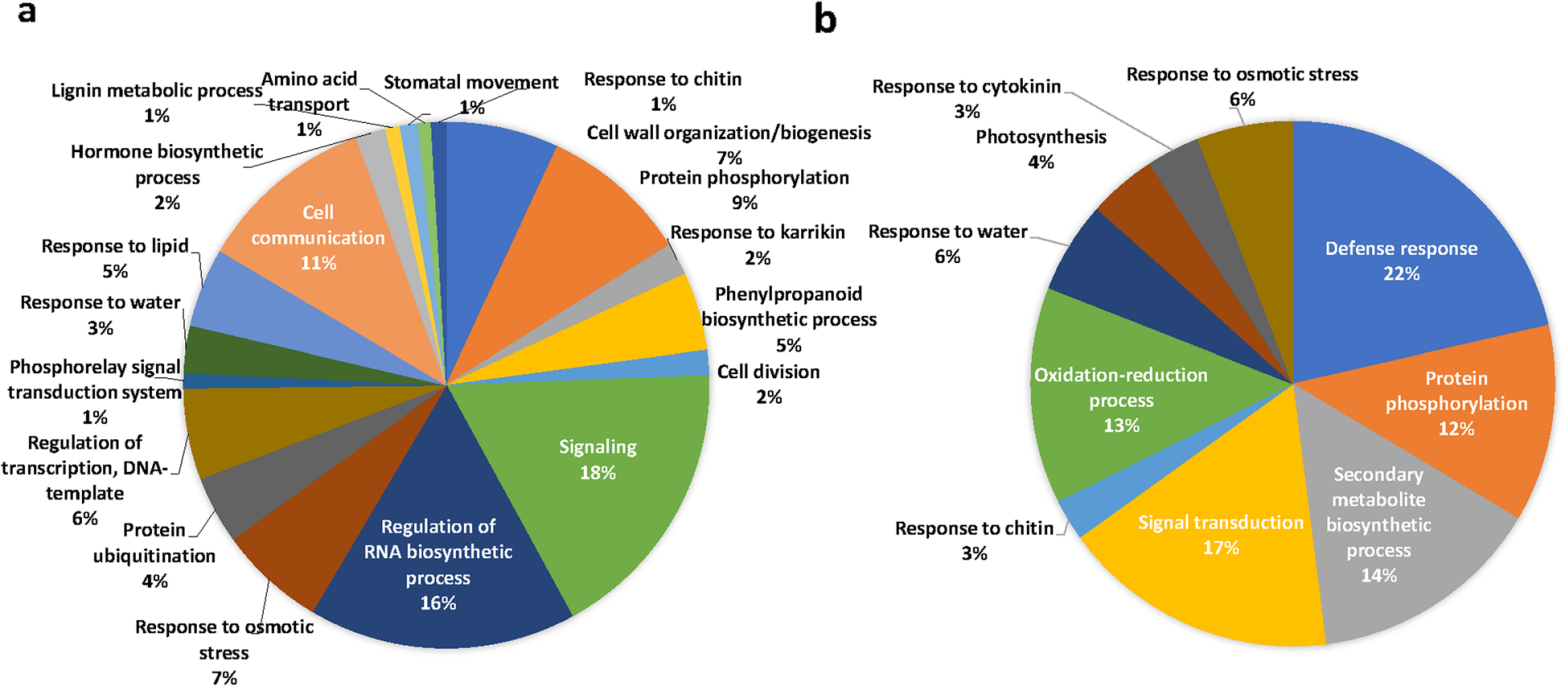
Gene ontology terms in biological process category for Si-induced drought tolerance in **a**) ILL 6002 and **b**) ILL 17537 for drought stress supplemented with Si Vs drought stress (DSi vs. D) comparison

**Fig. 9:**
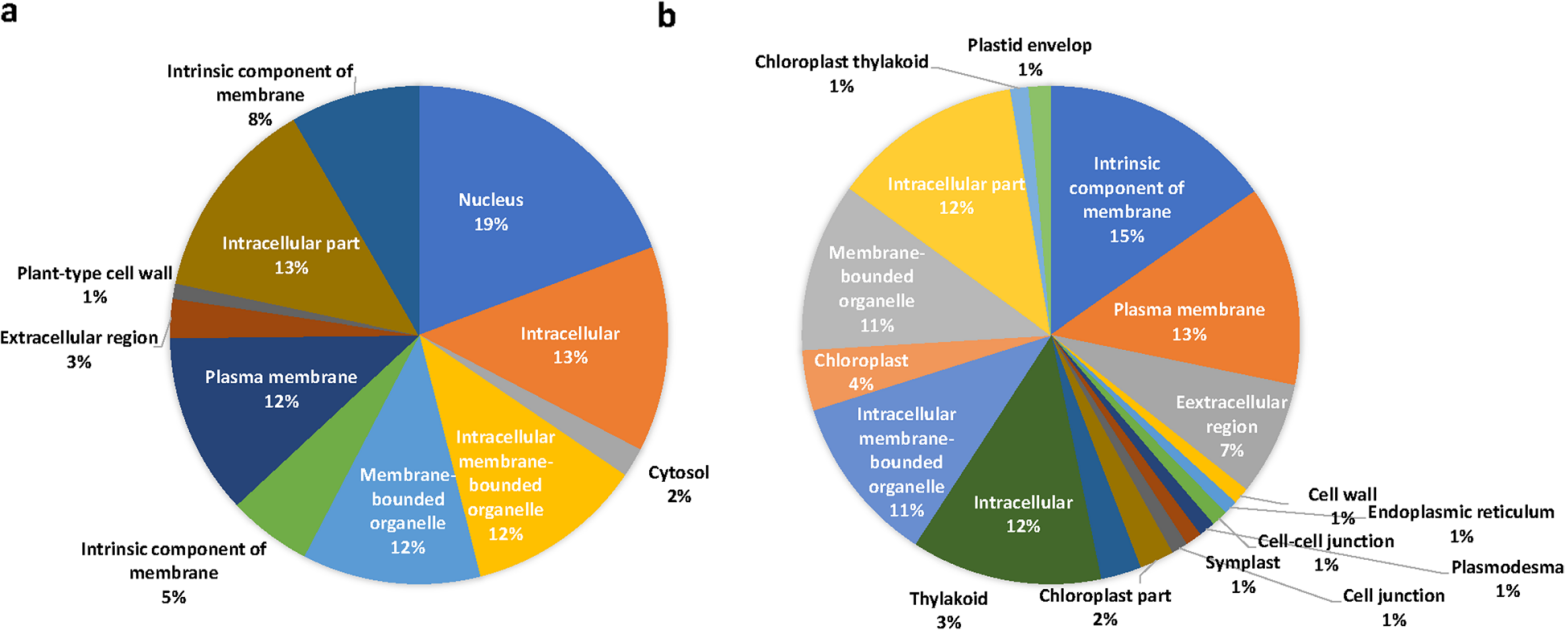
Gene ontology terms in cellular component category for Si-induced drought tolerance in (**a**) ILL 6002 and (**b**) ILL 17537 for drought stress supplemented with Si Vs drought stress (DSi vs. D) comparison

**Fig. 10:**
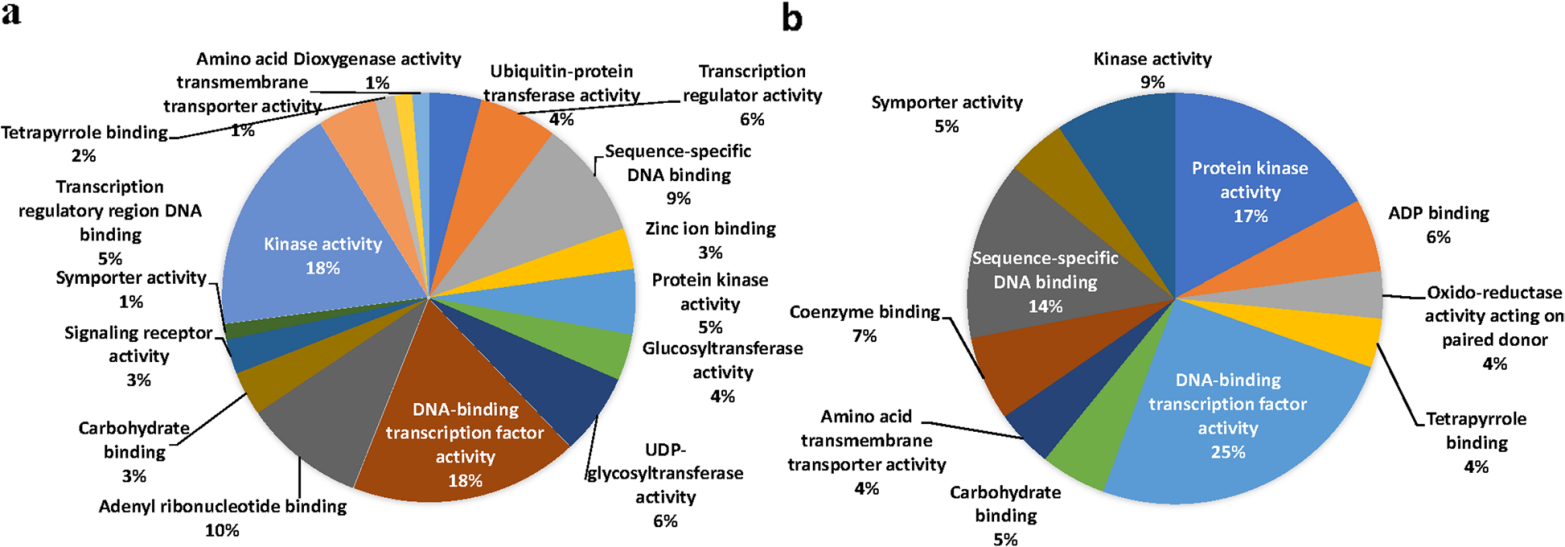
Gene ontology terms in molecular function category for Si-induced drought tolerance in (**a**) ILL 6002 and (**b**) ILL 17537 for drought stress supplemented with Si Vs drought stress (DSi vs. D) comparison

The functional annotation and gene ontology enrichment (GO) analysis was performed to categorize the top 100 upregulated and downregulated DEGs from both the genotypes during drought stress and non-stress conditions with Si supplementation. Gene ontology terms were classified into three principal categories: biological processes, cellular components, and molecular functions. GO enrichment analysis of both upregulated and downregulated DEGs in tissue samples led to recognising many GO terms in both tolerant and sensitive genotypes (Fig. 8). In the biological process category, the most represented and enriched categories were ‘signalling’, ‘regulation of RNA synthetic process’ and ‘cell communication’ in the drought-tolerant genotype, along with ‘protein phosphorylation’, ‘cell wall biogenesis/organization’, and ‘regulation of transcription’ (Fig. 9). However, genes associated with ‘defence response’, ‘signal transduction’, ‘secondary metabolite production’ and ‘oxidation–reduction process’ were the most enriched categories in the drought-sensitive genotype. For cellular components, the genes associated with 'intracellular', ‘plasma membrane', ‘intrinsic component of membrane’ and 'intracellular membrane-bounded organelle' were the most enriched categories in both genotypes. Furthermore, many other GO terms were also significantly enriched in the cellular component category (Fig. 10). Under the broad ‘molecular functions’ category, DEGs were significantly annotated for ‘DNA binding transcription activity’ and ‘protein kinase/kinase activity’, followed by ‘sequence-specific DNA binding’ and ‘carbohydrate binding’ in both genotypes (Fig. 10).

## Discussion

### Response of photosynthetic process, osmoprotective function and antioxidant metabolism related genes

As photosynthesis involves various organelles (stomata, chloroplast, photosynthetic pigments), systems and pathways (photosystems, the electron transport system, and CO_2_ reduction), any damage, at any level, caused by drought stress may reduce the overall photosynthetic efficiency of plants. The current study showed that Si increases photosynthetic efficiency under drought stress in lentil plants. The increase is attributed to the upregulation of genes related to stomatal and chloroplast movements, chlorophyll biosynthesis, photochemical quenching and gas exchange parameters (Table 4). These findings also validated our controlled and field-based research on Si-mediated drought stress tolerance in lentil plants, enhancing photosynthetic efficiency [54]. Our studies corroborate the findings of various researchers regarding Si’s positive role in mitigating drought stress by improving chlorophyll content and water use efficiency, while also protecting photosynthetic machinery from ROS. Additionally, it interacts with other physiological processes such as the absorption of macro and micronutrients and phytohormones, which also influence photosynthetic activity [55, 60–66]. Silicon demonstrated the capability to maintain irregular swelling and disintegrated thylakoid and chloroplast membranes [25, 52], altered stomatal aperture that influences the water uptake and water use efficiency [64, 67] and regulated the expression of photosynthetic genes (*PsbY, PsaH, PetC, PetH, Os09g26810, PetF, PsbP, PsbQ, PsbW* and Psb28) in plants during stress conditions, thus contributing to the efficient photosynthetic process. The present study’s findings reaffirm the role of Si in enhancing photosynthetic efficiency, as reported in numerous previous studies mentioned here.

In plants, ROS such as superoxide anion (O_2_^•−^), hydroxyl radical (•OH), hydrogen peroxide (H_2_O_2_) and singlet oxygen (1O^2^) play a vital role in the activation of stress-response networks, especially during stress conditions [68–71]. In this study, Si supplementation downregulated DEGs related to ROS (H_2_O_2_ and O_2_^.−^), revealing the crucial role of Si in ROS detoxification under stress (Table 4). Interestingly, Si application downregulated genes related to proline biosynthesis and metabolism in this study. In higher plants, proline is synthesized from glutamate mainly by the action of two enzymes: Δ1-pyrroline-5-carboxylic acid synthesis (P5CS) and pyrroline-5-carboxylic acid reductase (P5CR). Silicon-mediated downregulation of genes involved in proline synthesis and metabolism could be attributed to the accumulated proline-mediated feedback inhibition of P5CS, a rate-limiting enzyme for proline synthesis, consequently leading to downregulation of P5CR [72–76]. In the current study, the concentration of proline within the cells may have exceeded a certain threshold due to drought stress. With Si supplementation, the previously accumulated proline might have become bound to the active site of P5CS, consequently inhibiting its activity, leading to proline-mediated feedback inhibition in the cells. This result supports prior reports on Si-induced decrease in proline accumulation as a sign of plant stress injury alleviation [77, 78]. As the proline biosynthesis pathway is a conserved pathway, further studies could help identify the signal transduction pathways associated with proline biosynthesis, metabolism and the coordination of gene expressions, and other transcription factors under drought stress in response to Si supplementation.

Furthermore, upregulated genes related to the ‘glutathione metabolic process’ were identified in drought-stressed lentil plants in response to Si supplementation (Table 5). Glutathione is a non-enzymatic antioxidant playing a pivotal role in the ROS-scavenging strategies in plants [79]. The observed higher expression of genes related to photosynthetic process, osmoprotective mechanisms and antioxidant systems appears to be a common response among the two genotypes expressing Si-induced drought tolerance. The present findings strongly align with previous results of drought-stressed lentils, where Si application mitigated drought stress by regulating the accumulation of the metabolites mentioned above [7, 19].

### Phytohormones and Si-induced drought tolerance in lentil

Phytohormones (AUX, PA, ET, JA, ABA, GA, BR, and CK) are the key regulators in sensing and signalling numerous environmental stresses [80–83]. They induce drought stress tolerance in plants via synergistic and antagonistic interactions [84, 85]. Analysis of DEGs related to phytohormones, in the drought-stressed lentil genotypes in responses to Si, revealed that AUX, Karrikin (KAR), PA, ET and JA were the most important hormones involved in Si-mediated drought tolerance responses (Table 5). Silicon might have triggered the upregulation of DEGs related to AUX metabolism to develop a prolific root system in the plants, which is vital for drought tolerance. Previous reports also suggested that Si can accelerate the growth and development of the root system of drought-stressed plants [86]. Helaly’s [87] showed that Si supplementation increased AUX concentration in mango plants under drought stress. Silicon also upregulated the expression of an AUX-induced protein 5NG4-like gene (Csa6G104650), involved in the AUX signalling pathway, in salinity-stressed cucumber plants.

One novel finding of this study is the discovery of upregulated DEGs related to KAR response, under Si-mediated drought tolerance, in both genotypes. This is the first report of Si interaction with KARs under drought stress in plants. Karrikins are a class of butenolide compounds found in smoke promoting seed germination, seedling establishment and ecological diversity of plants [88, 89]. Karrikins have significant roles in mediating abiotic stress tolerance in plants due to their structural similarity with strigolactones [90, 91]. KAR enhanced drought tolerance in creeping bentgrass (*Agrostis stolonifera*) in association with antioxidative protection and regulation of stress-responsive gene expression [92]. Ma et al. [39] reported the upregulated expression of a secretory protein (33 kDa) related to KAR in rice plants under long-term Si-mediated Cd stress tolerance. A possible explanation for the observed upregulation of KAR in this study may be based on the previous experiments, where Si improved seed germination under drought stress in lentil [19]. It can be assumed that under drought stress, Si might have triggered the production of KARs, which interacted with other phytohormones and antioxidant compounds to induce a tolerance response in lentil plants. Li et al. (2017)’s [89] findings support these assumptions of KARs’ interaction with AUX and ABA, leading to the closure of stomata, activation of the antioxidant machinery and maintenance of oxidative homeostasis, in Arabidopsis under drought stress.

Polyamines are effective anti-senescence agents and ROS scavengers in plants [93]. Ethylene is a senescing hormone, whose inhibition can retard leaf senescence in plants [94]. Polyamines and ethylene share a biosynthetic association in terms of competitive demand for a limited pool of the common precursor, S-adenosyl methioninamine (SAM) [95]. Even though these two hormones share a common precursor, they act in antagonistic ways to senescence. The balance between these two opposite functions is crucial for one or the other adaptive strategy in plants and triggers their tolerance to various environmental stresses. Polyamines induce drought stress tolerance in plants by regulating genes encoding ABA biosynthesis enzymes [96].

The current study’s findings further confirm this balance and association between PA and ET through differentially regulated genes related to their biosynthesis in lentil genotypes under Si-mediated drought stress tolerance. These findings demonstrated that under drought stress Si upregulated genes associated with PA biosynthesis, which contributed to the efficient scavenging of ROS. Furthermore, under Si supplementation, downregulation of genes involved in ET production might have contributed to delayed senescence, with the help of upregulated genes for chlorophyll biosynthesis. This study strongly supports Biju et al.’s [19, 54] findings that Si supplementation effectively mitigated drought stress in lentil plants by regulating ROS and maintaining chlorophyll pigments under drought stress. Silicon application upregulated the expression of crucial ET biosynthesis genes (1-aminocyclopropane-1-carboxylic acid oxidase and 1-aminocyclopropane-1-carboxylic acid synthase) and reduced the expression of PA biosynthetic genes (Spermidine Synthase 1, Spermine Synthase, and Polyamine Oxidase 1), which resulted in an enhanced stress tolerance in tobacco plants [21, 97] However, this contradicts Manivannan and Ahn’s [27] and Yin et al. [26] reports on Sorghum plants where Si-mediated alleviation of salt stress was correlated with a decrease in ET biosynthesis. The interactions between ET and PAs imply that Si can influence ET production by regulating PA synthesis, thus maintaining the balance of PAs and ET biosynthesis.

Jasmonic acid is a positive regulator of drought stress in plants [98]. Allene oxide synthase (AOS) and allene oxide cyclase (AOC) are enzymes in the octadecanoid pathway that lead jasmonic acid and biosynthesis.The current study identified downregulated genes related to the activities of AOS and AOC and metabolism of JA-mediated signalling pathways. These findings demonstrate that Si might have triggered the crosstalk interactions of JA with other phytohormones leading to increased drought stress tolerance. The results support Hamayun et al.’s [99] findings in drought-stressed soybean and Kim et al.’s [49] in rice under heavy metal stress, where JA synthesis was negatively affected in response to Si treatment. Furthermore, Si-induced negative regulation of JA can also be explained partially through Dhakarey et al.’s [100] studies, which suggested that JA might be a negative regulator of drought stress tolerance in rice.

Abscisic acid is a plant stress hormone crucial for plant growth and development. ABA significantly integrates various stress signalling pathways and controls downstream stress responses [101]. This study identified upregulated DEGs for the biosynthesis of ABA and the activity of abscisic acid 8'-hydroxylase, an enzyme responsible for the first step in the oxidative degradation of ABA [102]. This demonstrates the role of Si in maintaining ABA homeostasis in lentils under drought stress. Likewise, downregulated DEGs were found for the negative regulation of the ABA-activated signalling pathway, indicating the regulatory role of Si in modulating ABA responses under drought stress. These results support previous experimental results where Si improved seed germination in lentil under polyethylene glycol (PEG) induced drought stress [19] since the seed germination processes are regulated by ABA and GA homeostasis [103]. Kim et al. [48] also found that Si enhanced the expression of ABA-biosynthetic genes, Zeaxanthin epoxidase (ZEP) and 9-cis-epoxycarotenoid oxygenase 1 and 4 (NCED1, NCED4) in salt-stressed rice, by showing an antagonistic relationship between ABA and Si.

Gibberellins are essential phytohormones known for their role in plant internode elongation [104]. Silicon is also known for altering endogenous GA levels in plants under stress and non-stress conditions [41, 48, 49, 105]. From the current study, it seems possible that Si might have affected lentil shoot growth and shoot proliferation under drought stress by upregulating the expression of genes related to GA synthesis and signalling pathways. These results agree with Hamayun et al.’s [99] and Lee et al.’s [106] findings, where they reported a higher accumulation of GA levels upon Si addition in soybean under drought and salt stress, respectively. Furthermore, upregulation of a protein related to GA (gibberellin 20 oxidase), involved in the gibberellin signalling pathway was found in salt-stressed tomato plants in response to Si supply [107].

Maintaining a suitable concentration SA concentration in plants can alleviate various stresses by regulating different biochemical pathways [108]. Downregulated DEGs were found for SA metabolic processes in the drought-tolerant genotype, while upregulated DEGs related to the SA biosynthetic process were found in the sensitive genotype. Differential regulation of the phenolic phytohormone, SA, in response to Si in both the genotypes might be related to the concentration of SA accumulated in cells and different drought tolerance levels of the genotypes. There are reports on decreased endogenous SA content in soybean plants, grown under drought stress [99] and downregulated DEGs encoding SA-binding proteins under biotic stress in tomato plants [109]. response to Si supplementation. However, no studies have compared SA accumulation and expression levels in drought-tolerant and sensitive plants in response to Si application. Moreover, additional upregulated DEGs for BRs and downregulated DEGs for CK synthesis were found only in the drought-sensitive genotype (ILL 7537), further suggesting a detailed investigation of the role of Si in regulating the phytohormonal interactions in plants under drought stress via comparison in drought-tolerant and sensitive genotypes is required.

### Silicon triggers cell wall development, and vasculature biogenesis

Silicon supplementation of drought-stressed lentil genotypes modulated the expression of genes related to cell wall development and vasculature biogenesis (Table 6). This finding provides strong evidence for the possible interaction or the binding of polymerized Si with cell wall components as investigated in previous studies [110–113]. These findings also support Si-dependent strengthening and reinforcement of the cell wall, as a protective adaptation strategy in plants, especially in the dicots, which are low Si accumulators compared with the monocots. Furthermore, these results strongly re-establish the role of Si in improving the mechanical properties and regeneration of cell walls in plants [113–115]. Lignin is the most abundant structural polymer found in the plant cell walls, after cellulose. The presence of Si is reported in plant epidermal cell walls associated with lignin-carbohydrate complexes [110]. These results are consistent with Si-induced enhancement of lignin deposits in roots of salinity-stressed rice plants and the formation of silica bodies in tall fescue (Festuca arundinacea) and bentgrass (Agrostis stolonifera) plants [116, 117]. Silicon also improved the lodging resistance of rape (Brassica napus) stems by improving lignin accumulation and the mechanical tissue structure [28]. Furthermore, Si is also known to enhance lignin accumulation in plants under biotic stresses [118, 119].

Vascular tissues (xylem and phloem) provide mechanical strength and facilitate the transport of water, nutrients, hormones and other signalling molecules throughout the plant. Even though the formation of the vascular system is a well-organized developmental process in plants, it can also be flexible in response to environmental changes [120]. Plant hormones, peptide signalling, and transcriptional regulators are known to regulate the development and patterning of the xylem and phloem in plants [119]. Based on the current findings, it can be inferred that Si plays a crucial role in maintaining the structural components and regulating the source to sink transport in lentil via maintaining phytohormonal homeostasis under drought stress. The role of Si in the development and differentiation of vascular tissues certainly needs to be addressed.

### Carbohydrate and lipid metabolism in response to Si supplementation

Silicon supplementation significantly regulated the genes related to carbohydrate and lipid metabolism in lentil plants under drought stress (Table 7). These results suggest that added Si might have positively regulated the loading and unloading of sucrose via phloem by maintaining homeostasis between starch and sucrose levels as a protective adaptation strategy under drought stress in plants. These results differ from Yin et al.’s [26] findings in drought-stressed sorghum; however, they are consistent with the findings of Zhu et al. [34], who noticed an increase in starch and a decrease in sucrose contents in cucumber under salinity stress with Si treatment. Silicon-induced upregulation of trehalose metabolism could also be a part of the plant’s adaptive strategy to combat drought stress. These results support the findings of Manivannan et al. [121], who reported Si mitigated salinity stress in capsicum by regulating the expression of proteins and carbohydrate metabolism.

The downregulated DEGs for oxidation of unsaturated fatty acids indicate reduced lipid peroxidation, preventing cell membrane damage under drought stress [122]. Previous results demonstrated that Si mitigated drought stress by reducing lipid peroxidation in lentil plants under similar experimental conditions [7]. Furthermore, Si might have also maintained the optimal membrane fluidity to prevent structural and functional deterioration of cell membrane by downregulating the lipid concentrations to a minimum level under drought stress. Thus, it can be inferred that Si might have some regulatory effects on lipid composition and the degree of fatty acid unsaturation in plants under stress. Several plant studies have also reported Si-induced drought tolerance via reduced lipid peroxidation [16, 61, 77, 78].

### Silicon modulated the expression of genes related to the metabolism of amino acids, secondary metabolites and drought recovery

Silicon triggered the expression of genes related to protein phosphorylation and protein kinase activity in lentil plants under drought stress (Table 8). Protein kinases act to phosphorylate and dephosphorylate their targets (proteins/amino acids), thereby maintaining drought-signalling homeostasis in plants [123, 124]. Ubiquitin plays a key role in plant hormone synthesis, hormonal signalling cascades and other defence mechanisms [125]. Downregulation of the ubiquitin protein ligase and ubiquitin protein transferase activity by Si, in drought-stressed lentil plants, might have contributed to the fine adjustment of hormonal signalling pathways and other defence mechanisms at the molecular level. The ability of Si to modulate the expression of proteins involved in the ubiquitin-mediated nucleosome pathway was identified in salt-stressed capsicum [121]. Although this study cannot rule out the differential regulation of protein and amino acid metabolism in response to Si under drought stress for both genotypes, it is suggested that Si might have maintained amino acid homeostasis by regulating the activity of protein kinases to enhance drought stress tolerance. These results corroborate the findings of Pereira et al. [126], where a positive corrripathielation was found between increased amino acid contents and osmotic adjustment in response to Si in drought-stressed capsicum. Silicon triggers vital secondary metabolite biosynthetic pathways in plants, especially under stress conditions [127]. Results from the present study suggest that Si may also act as a regulatory molecule under drought stress to protect the plant by effectively synthesizing secondary metabolites in drought-sensitive genotypes (Table 9). This result supports our previous findings, where Si supplementation increased the accumulation of flavonoids in drought-stressed lentil plants [8]. It also indicates the existence of genetic differences among different plant genotypes and species for the regulatory mechanism related to secondary metabolite synthesis and their role in stress tolerance. Furthermore, the role of Si as a ‘drought stress alleviator’ in lentils is confirmed by the findings of differentially regulated genes related to drought tolerance recovery in both the genotypes under Si-mediated drought tolerance (Table 10). Upregulation of genes involved in water homeostasis validated all other findings in this study.

### Differential expression of genes related to biological processes, cellular components and molecular functions as revealed by gene ontology (GO) annotation

The findings shown in Figs. 8, 9 and 10 indicate Si’s crucial role in regulating all the biological processes in lentil genotypes, to alleviate the adverse effects of drought stress. Results of the cellular component category demonstrate that Si might act as a signal molecule in regulating cell metabolism and maintaining the structural integrity of cells and membranes under drought stress, as He et al. [128] (2015) suggested in rice plants. The findings from this study are well supported by previous work done in this area, such as polymerized Si accumulation in the epidermal cell walls of rice [129], and high root endodermal silicification in sorghum [130], under Si-mediated drought stress tolerance. Moreover, many reviews are also available on the protective role of Si on cell walls and membranes in plants under environmental stresses [20, 21]. The molecular category results further confirm the involvement of Si in phosphorylation, active transport of ions and its binding with other cellular molecules to lessen cell or membrane damage during drought stress (Fig. 10). The GO terms related such as ‘chloroplast’, “thylakoid’ and ‘plastid envelop’ demonstrate the active engagement of Si in photosynthetic processes. A recent study in rice also showed that Si improved the photosynthetic performance by maintaining thylakoid membrane protein components such as PSI core binding LHCI (Light harvesting complex I), PSI core, F1-ATPase binding Cytb6/f complex, PSII core, trimeric LHCII and monomeric LHCII, under drought stress [52]. These results corroborate the observations of Kang et al. [131] in the succulent xerophytic plant, *Zygophyllum xanthoxylum*, where they found this C3 plant accumulated high amounts of Si and utilized it as an osmoregulator to improve photosynthetic activity and antioxidant enzyme activities under drought stress.

## Methods

### Plant materials and drought stress treatments

Two lentil genotypes, ILL 6002 (drought tolerant) and ILL 7537 (drought sensitive) were selected as experimental materials and the seeds were procured from The Australian Grains Genebank (AGG), Horsham, Victoria. These genotypes were identified as drought tolerant and drought sensitive from a previous drought stress tolerance screening experiment [6]. This experiment was conducted in a growth room (temperature: 23 ± 2 °C; relative humidity: 45–50%; photoperiod: 12 h; light intensity: 300–325 μmol m^2^ s^−1^ from metal halide illumination lamps (MH 400 W/640 E40 CLU 1SL/6, Netherlands) of the University of Melbourne, Parkville. Lentil seeds were sown, after surface-sterilisation (30% [v/v] hydrogen peroxide solution), in 950 mL plastic pots filled with 700 g lentil potting mix (70% garden soil and 30% composted pine bark with 1.6 kg dolomite lime per 60 L potting mix, pH 7.00). The source of silica is sodium metasilicate (Na_2_SiO_3_) and 2 mM of Na_2_SiO_3_ solution (500 ml kg^−1^ potting mix) was added to the pots before sowing seeds. Silicon is soluble in the soil only at pH < 9 and concentration at or below 2 mM [132–135]. Our previous experiments also showed that 2 mM Si improved the drought stress tolerance in lentil plants [6–8, 19]. Therefore, this experiment was designed with 2 mM of Si solution. The molarity of Na_2_SiO_3_ was calculated based on the potting mix volume and solution’s final pH was made to 7.5 using 0.1 N hydrochloric acid. Plants were fertilised with Nitrosol (Amsgrow) during the vegetative stage to maintain normal plant growth. The experiment was carried out as a completely randomized design in three replicates with two lentil genotypes and four treatments. Lentil plants were given severe drought stress (20% field capacity) in this study as our previous research showed that maximum plant damage occurs at severe drought stress (Biju et al. 2018; 2021a). The treatments were as follows: (i) control (C -well watered, 100% FC), (ii) severe drought stress (D-20% FC), (iii) severe drought stress with supplemented Si (DSi), and (iv) Si alone (Si). The control treatments were supplied with sodium sulphate (Na_2_SO_4_; 2 mM) to balance the sodium levels in treatments (ii) and (iv) supplied with Si. Drought stress was imposed at the reproductive growth stage (R1 stage-anthesis) and continued for 28 days at respective field capacities. Furthermore, to maintain uniformity of growing conditions and elimination of light and air flow stress biases, pots were reorganised weekly in the growth room. Leaf samples for RNA analysis were harvested at the end of the drought stress treatment period when the plants reached the R3 stage (pod development stage). The growth stages of the plants were assessed using the descriptors for stages of development in lentil plants [136].

### RNA isolation, cDNA library preparation and Illumina sequencing

Total RNA from leaf samples of various treatments were extracted using RNeasy plant mini kit along with DNase treatment, according to the manufacturer’s instruction (Qiagen, USA). The quantification of RNA was determined using a NanoDrop ND8000 (Thermo Scientific, USA). The integrity and quality control check for RNA was done on the tape station and the Agilent bioanalyzer (Agilent Technologies, Inc., Santa Clara, CA, USA). The samples were normalised to an input RNA weight of 1 µg for processing. The samples underwent high-throughput sequencing on the Illumina HiSeq platform at the Australian Genome Research Facility (Melbourne, Australia). RNA seq libraries were constructed using the illumina TruSeq Stranded mRNA kit, following the manufacturer’s instructions (Illumina, USA). The RNA seq experiment (including library preparation) was completed with three biological replicates (only 2 replicates for S24, which failed the library preparation). Image analysis was performed in real-time by the HiSeq Control Software (HCS) v2.2.68 and real-time analysis (RTA) v1.18.66.3, running on the instrument’s computer. The RTA performs real-time base calling on the HiSeq instrument computer. Then the Illumina bcl2fastq 2.20.0.422 pipeline was used to generate the sequence data.

### Mapping of RNA-Seq reads and differential gene expression analysis

*Lens culinaris* genome v1.2 and annotation v1.2b were used for the analysis [137]. Transcripts were extracted using gffread v0.9.10 (https://github.com/gpertea/gffread). The reads were pseudo aligned and transcript abundance was estimated using Kallisto v0.44.0 [138]. The estimated counts were loaded, and differential gene expression analysis was performed using DEApp (https://gallery.shinyapps.io/DEApp/) [139]. The Raw Count Data file containing summarized count results of all samples in the experiment, and the Meta-data Table file containing summarized experimental design information for each sample were used as input data. Low expression genetic features were removed after alignment if the count per million (CPM) value was ≤ 1 in less than two samples. After filtering out the low expression genomic features, the samples’ normalization and multidimensional scaling (MDS) plots were also obtained to illustrate the samples’ distribution and relationship. DE analysis was performed on the raw count data from all the treatments using edgeR, using cut‐off values of log Folds Change (log FC)—1.5 and a false discovery rate (FDR) adjusted to a *P* value < 0.05. A dispersion plot, overall DE analysis results, and statistically significant DE results were generated, together with a volcano plot related to the specified parameters and cut-off values. Venn diagrams were created with differentially expressed genes (DEGs) for each comparison and all the possible combinations from the drought-tolerant ILL 6002 and the drought-sensitive ILL 7537, using the website (http://bioinformatics.psb.ugent.be/webtools/Venn/). The Heatmapper program (http://www.heatmapper.ca/) was used to draw the heatmap of the significant DEGs, in response to various treatments. All the raw data is deposited in Github repository https://github.com/SajithaBiju/Data-LentilSilicon.

### Functional annotation and Gene Ontology (GO) enrichment

The DEGs were annotated for gene ontology (GO) terms and categorized into Molecular Function (MF), Cellular Component (CC), and Biological Process (BP) categories. GO enrichment was performed using top GO v2.34.0 [140] using the ‘weight’ method to adjust for multiple comparisons. *Lens culinaris* genes were annotated with GO terms by transferring *A. thaliana* GO annotation (ATH_GO_GOSLIM.txt; https://www.arabidopsis.org/) to the best *L. culinaris* match as established by BLASTP v2.6.0 [141] comparison (-max_target_seqs 1 -num_threads 16 -evalue 1e-5 -outfmt '6 qseqid sseqid pident length mismatch gapopen qstart qend sstart send evalue bitscore qcovs qlen'). The GO enrichment (p value ≤ 0.05) was investigated by subjecting all DEGs to the GO database (http://www.geneontology. org/) to further classify genes, or their products, into terms of molecular function, biological process and cellular component which helps understand the genes’ biological functions (https://github.com/SajithaBiju/Data-LentilSilicon).

## Conclusion

**Fig. 11:**
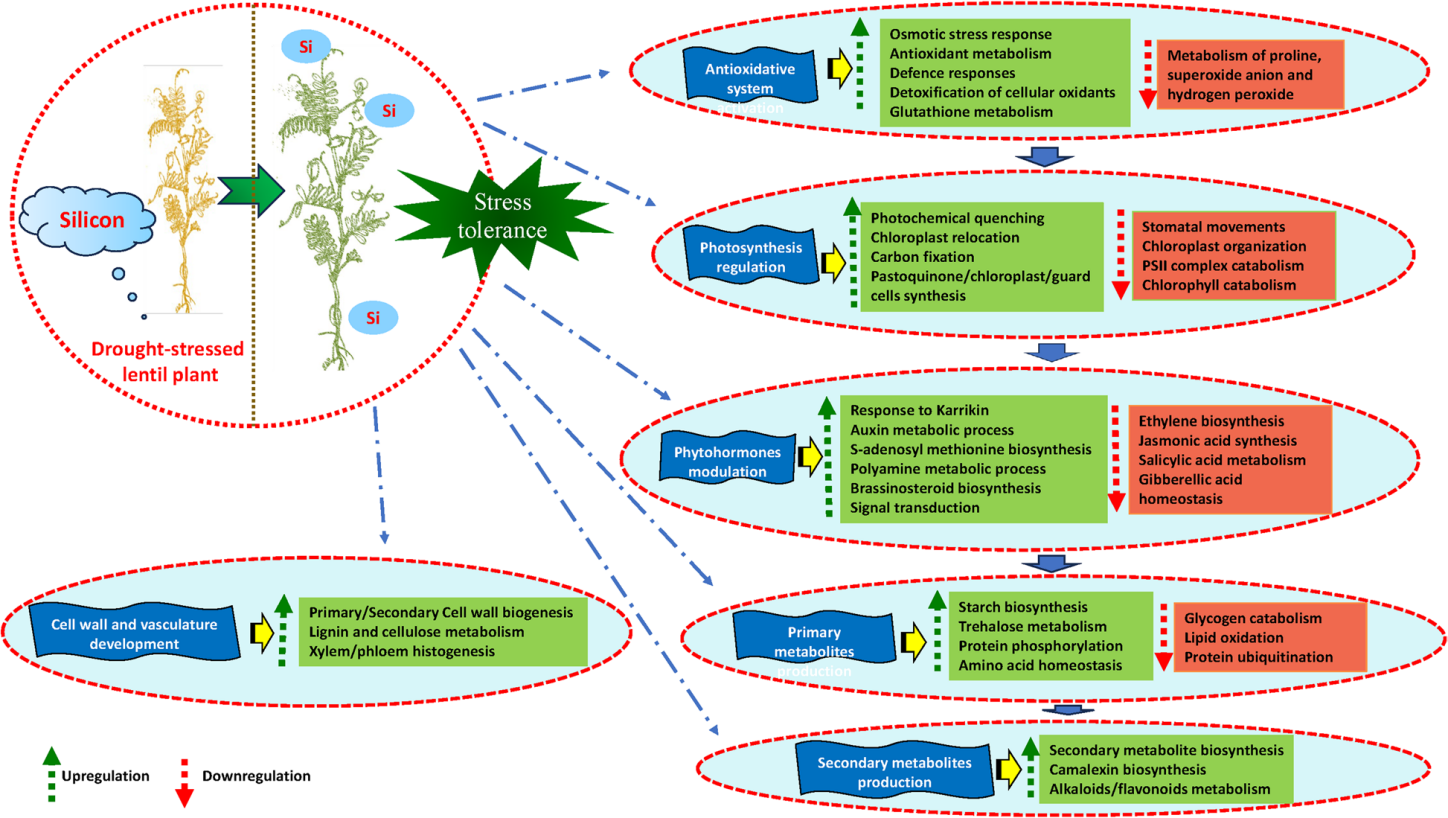
Crosstalk between different biochemical pathways in silicon-mediated drought stress responses in lentil plants

The current study determined the role of Si in mitigating drought stress in two lentil genotypes employing RNA sequencing to identify biological cellular and molecular pathways in continuation of the physiological and biochemical experiments undertaken in lentils under Si-mediated drought tolerance. The results have provided considerable evidence to demonstrate that lentil adaptation to drought stress is a diverse approach involving the modulation of the expression of several genes that regulate various metabolic functions like photosynthesis, antioxidant defence system, osmotic balance, hormonal regulation and crosstalk, signalling, amino acid biosynthesis, carbohydrate and lipid metabolism and other defence related pathways that assist lentil in drought tolerance recovery (Fig. 11). This study has provided novel insights into plants’ responses to drought and new leads for functional studies of genes involved in Si-induced drought tolerance. Furthermore, the findings confirmed that additional protective strategies are induced in sensitive genotypes compared with the tolerant genotype under Si-mediated drought tolerance, as evidenced by identifying upregulated DEGs related to secondary metabolite synthesis. The differential upregulation noticed in the hormonal cascade prioritises the need for a detailed exploration to unveil the role of Si in maintaining hormonal homeostasis under drought stress. Furthermore, this data suggests that the studies focussing on Si-induced drought tolerance in open field conditions should also focus on the interaction of Si with the synthesis and development of cell walls and vascular tissues. These findings imply that further bioinformatics and metabolomics should be conducted to extricate the complex control networks involved in Si-mediated drought tolerance in plants. With a better understanding of the role and mechanism of action of Si in stress responses, it would be possible to develop new breeding or Si-mediated stress management strategies to enhance plant survival in adverse environmental conditions.

## Data Availability

All data generated or analysed during this study are included in this published article and raw data are provided in Github repository https://github.com/SajithaBiju/Data-LentilSilicon.

## Abbreviations

ABA: Abscisic acid
ATAF: *Arbidopsis thaliana* Activating factor
AUX: Auxin
BR: Brassinosteroid
Ci: Intercellular CO_2_ concentration
CPM: Count per million
CUC: Cup-shaped cotyledon
CK: Cytokinin
DEGs: Differentially expressed genes
*DREB2A*: Dehydration responsive element-binding protein
ET: Ethylene
ETR: Electron transport rate
Fv/Fm: Maximum photochemical efficiency of PSII
GA: Gibberellic acid
GO: Gene ontology
gs: Stomatal conductance
JA: Jasmonic acid
KAR: Karrikin
MDS: Multidimensional scaling
NAM: No apical meristem
NPQ: Non-photochemical quenching
PA: Polyamine
Pn: Net photosynthetic rate
qP: Photochemical quenching
RIN: RNA integrity number
RNA Seq: RNA Sequencing analysis
ROS: Reactive oxygen species
SA: Salicylic acid
Si: Silicon
TF: Transcription factors
Tr: Transpiration rate
